# A novel ATG5 interaction with Ku70 potentiates DNA repair upon genotoxic stress

**DOI:** 10.1038/s41598-022-11704-9

**Published:** 2022-05-17

**Authors:** Sinem Demirbag-Sarikaya, Yunus Akkoc, Sıla Turgut, Secil Erbil-Bilir, Nur Mehpare Kocaturk, Joern Dengjel, Devrim Gozuacik

**Affiliations:** 1 SUNUM Nanotechnology Research and Application Center, 34956 Istanbul, Turkey; 2 Koç University Research Centre for Translational Medicine (KUTTAM), 34010 Istanbul, Turkey; 3 Department of Biology, University of Fribourg, Chemin du Musée 10, 1700 Fribourg, Switzerland; 4 Koç University School of Medicine, 34010 Istanbul, Turkey

**Keywords:** Cell biology, Molecular biology

## Abstract

The maintenance of cellular homeostasis in living organisms requires a balance between anabolic and catabolic reactions. Macroautophagy (autophagy herein) is determined as one of the major catabolic reactions. Autophagy is an evolutionarily conserved stress response pathway that is activated by various insults including DNA damage. All sorts of damage to DNA potentially cause loss of genetic information and trigger genomic instability. Most of these lesions are repaired by the activation of DNA damage response following DNA repair mechanisms. Here we describe, a novel protein complex containing the autophagy protein ATG5 and the non-homologous end-joining repair system proteins. We discovered for the first time that ATG5 interacted with both Ku80 (*XRCC5*) and Ku70 (*XRCC6*). This novel interaction is facilitated mainly via Ku70. Our results suggest that this interaction is dynamic and enhanced upon genotoxic stresses. Strikingly, we identified that ATG5-Ku70 interaction is necessary for DNA repair and effective recovery from genotoxic stress. Therefore, our results are demonstrating a novel, direct, dynamic, and functional interaction between ATG5 and Ku70 proteins that plays a crucial role in DNA repair under genotoxic stress conditions.

## Introduction

The balance between anabolic and catabolic reactions is required for the maintenance of cellular homeostasis. Autophagy is an evolutionarily conserved catabolic reaction, and its activation serves as a cellular clearance mechanism from yeast to mammals^1–3^. Upon autophagic stimuli, multi-step processes occur in the cell, including the formation of doubled-membrane structure, maturation of autophagic vesicles and fusion with lysosomes which eventually resulted in the degradation of cargo molecules. Autophagy facilitates the elimination of the damaged organelles and leads to the production of new building blocks, thereby functioning as a pro-survival mechanism^4–7^.

So far, more than 30 different core autophagy proteins have been identified^8^. The autophagy process requires the involvement of those core proteins and their orchestration. Especially ATG5 protein plays a critical role during elongation of the autophagosome. First, ATG5 conjugates with ATG12 via its Lys 130 residue. ATG16L1 then further conjugates with established ATG12-ATG5 conjugate^9,10^. Then, this complex acts like an E3-ubiquitin ligase and facilitates the lipidation of ATG8 (MAP1LC3 or shortly LC3 in mammals). Lipidated LC3 (LC3-II) localizes on autophagosome and forms dot-like structures^11^. Concomitantly, the loss of ATG5 triggered an autophagic catastrophe that suggests its necessity for the autophagy process^12^.

Maintenance of genomic stability is ensured by a synchronized DNA damage response (DDR) mechanism patrolling for detection, signaling and repair of DNA lesions. Although single-strand breaks (SSBs) occur more extensively than double-strand breaks (DSBs) in the genome, DSBs are more lethal and immediate repair of DSBs is indispensable for genomic integrity and cellular survival.

DSBs are primarily restored by two different mechanisms including homologous recombination (HR) and non-homologous end-joining (NHEJ)^13,14^. Several proteins including Ku70, Ku80 and a DNA-dependent protein kinase catalytic subunit (DNA-PKcs) etc., and their activations are involved in NHEJ to provide chromosomal integrity^13,15^. Interaction of Ku70 (*XRCC6*) and Ku80 (*XRCC5*) initiates NHEJ where damage happens. This heterodimer act as a flag on the lesion and through recognition of DNA-PKcs, they facilitate the recruitment of further complex components to finalize the repair^16^.

The integrity of the human genome is threatened by a range of exogenous and endogenous agents that can result in damage to DNA, thereby disrupting cellular homeostasis. As a cellular stress response mechanism, autophagy plays a crucial role during genotoxic stress conditions^17^. Altered autophagic activity has been reported in the presence of DNA damage. Moreover, several individual autophagy proteins have been associated with DNA damage and DDR. Yet, the role of autophagy in the NHEJ DNA repair mechanism and its contribution during genotoxic stress has remained elusive^18^.

In this study, we observed that ATG5 interacts with several NHEJ components under basal and genotoxic stress conditions. We identified a novel and direct interaction between ATG5, autophagy protein, and Ku70, an NHEJ repair mechanism protein. ATG5-Ku70 interaction predominantly occurs in the nuclei. This interaction is dynamic and responds to genotoxic stress conditions e.g., etoposide, cisplatin and doxorubicin. We also observed that this interaction is required for an effective DNA repair. Strikingly, loss of ATG5 accumulated unrepaired DNA lesions following exposure to damaging agents. Repair attenuated cells were manifested proliferation disability during recovery. Concomitantly, restoring the ATG5 protein was sufficient to restore the DNA repair capacity of the cells.

Hence, our study introduces for the first time a novel interaction partner of ATG5 and associates the interaction with the effective DNA repair capacity of the cells following genotoxic stress.

## Results

### Autophagy protein ATG5 Interacts with the NHEJ components

**Figure 1:**
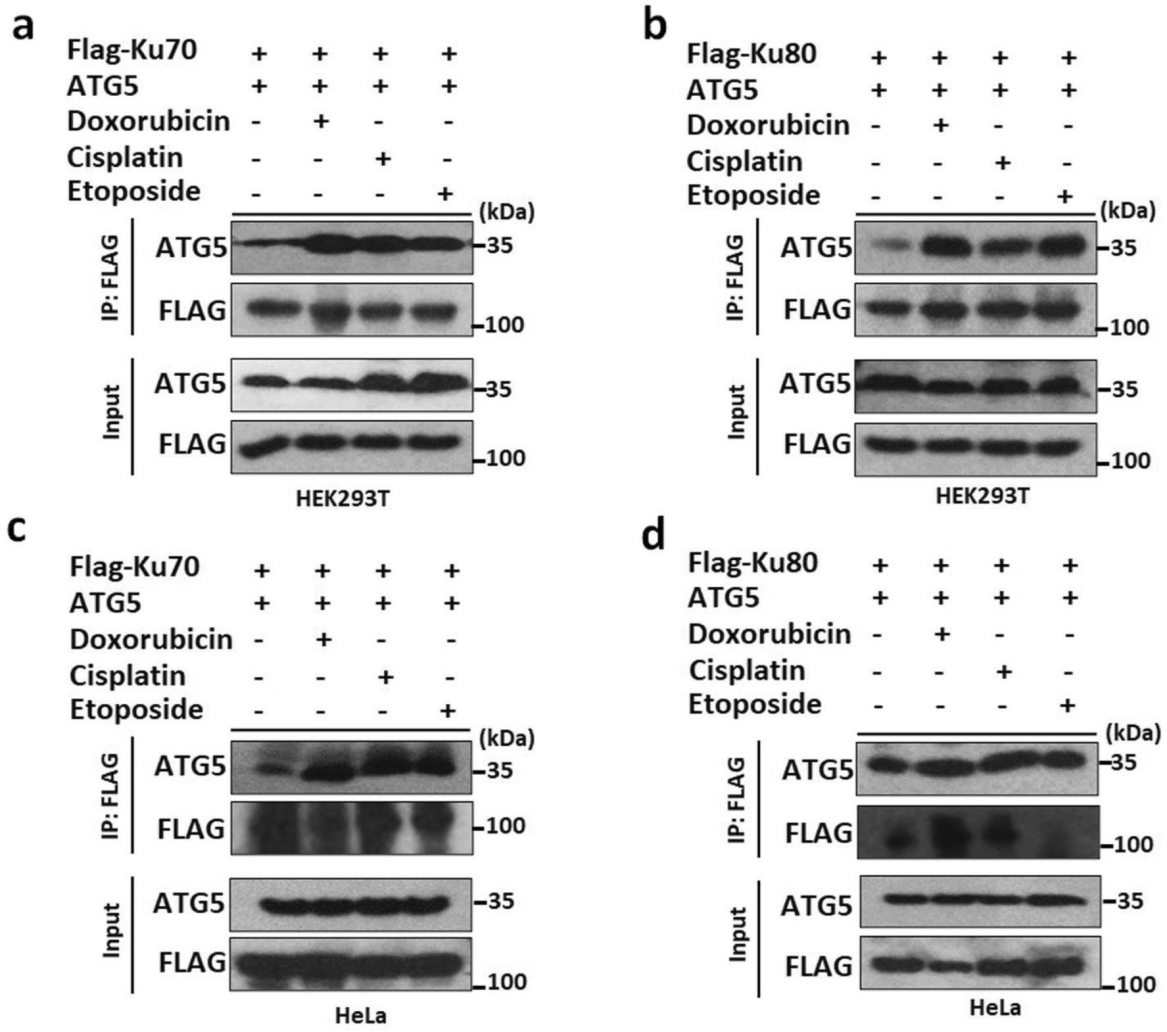
NHEJ components Ku70 and Ku80 are novel ATG5 interactors. (**a**) HEK293T cells were co-transfected with plasmids encoding FLAG-tagged Ku70 and/or non-tagged full-length ATG5 proteins. (**b**) HEK293T cells were co-transfected with plasmids encoding FLAG-tagged Ku80 and/or non-tagged full-length ATG5 proteins. (**c**) HeLa cells were co-transfected with plasmids encoding FLAG-tagged Ku70 and/or non-tagged full-length ATG5 proteins. (**d**) HeLa cells were co-transfected with plasmids encoding FLAG-tagged Ku80 and/or non-tagged full-length ATG5 proteins. Cells were exposed to Etoposide, Doxorubicin and Cisplatin after 24 h post-transfection. 50 µM Etoposide, 12.5 µg/ml Cisplatin, 1 µm Doxorubicin; 25 µM Etoposide, 1 µg/ml Cisplatin, 100 nm Doxorubicin were used for HEK293T and HeLa cells, respectively. 48 h later, IPs were performed using FLAG beads. Anti-ATG5 and anti-FLAG antibodies were used for immunoblotting. Input, total cell extract. Molecular Mass was shown in kilodaltons (kDa).

To discover novel ATG5 interactors and autophagy-related proteins, we performed Tri-SILAC labeling and LC–MS/MS analysis using a Flag-tagged ATG5 protein isolated from HEK293T and HeLa cells^19^. SILAC–LC–MS/MS analysis was used as ATG5 bait and revealed the interactions with both Ku70, Ku80 and DNA-PKcs in both cell lines (Supplementary Fig. 1a–c). We confirmed the interaction of Ku70 and Ku80 through performing co-immunoprecipitation (Co-IP) experiments in HEK293T (Fig. 1a,b) and HeLa (Fig. 1c,d) cells following overexpression of both full-length proteins. Strikingly, we observed that the interaction prominently was increased upon exposure to genotoxic stress conditions including etoposide, doxorubicin and cisplatin in both HEK293T and HeLa cells.

DNA-dependent protein associations were selectively inhibited by ethidium bromide (EtBr) in the precipitation reaction without any evident effect on DNA-independent protein associations. As Ku70 and Ku80 both are DNA binding proteins we performed the same Co-IPs in the presence of ethidium bromide. EtBr did not alter any of the interactions with ATG5 which suggest DNA-independent protein association (Supplementary Fig. 2a–d). ATG5 protein predominantly was found to interact with ATG12 for the elongation of autophagosome under physiological conditions^10^.

**Figure 2:**
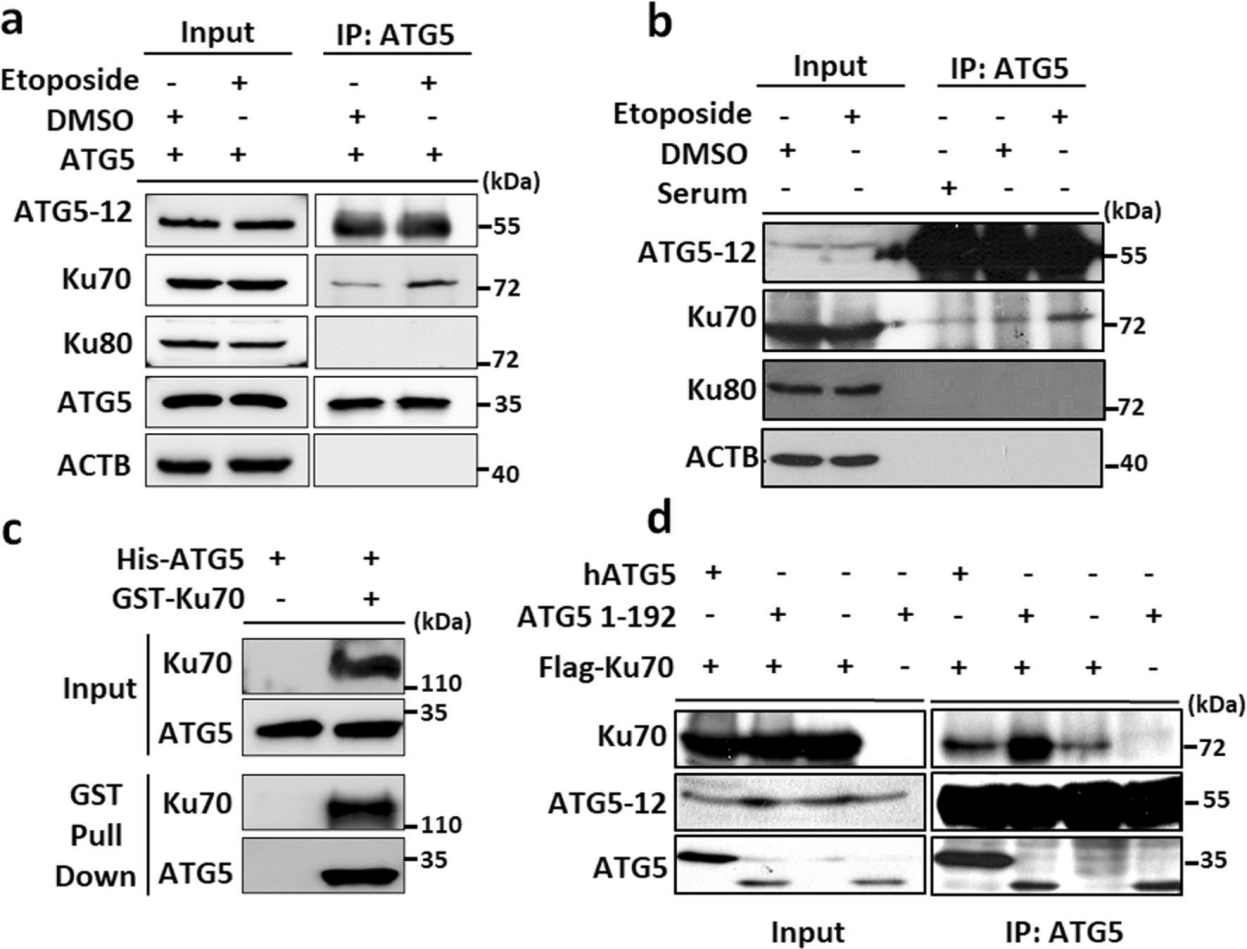
ATG5 endogenously interacts with Ku70. (**a**) HEK293T cells were transfected with plasmids encoding non-tagged ATG5. Cells were exposed to either Etoposide or DMSO, as a vehicle, and IPs were performed with the anti-ATG5 antibody at 48 h post-transfection. (**b**) Endogenous IPs were performed from HEK293T cells treated with Etoposide for along 24 h. DMSO was used as a vehicle. Rabbit serum was used as a stickiness control. (**c**) GST Pull-down assay was performed. Glutathione-Sepharose beads were bound to GST-Ku70 recombinant protein or not incubated with His-ATG5 recombinant protein and washed (Input: Immunoblotting of recombinant proteins; GST-pull down: proteins following pull-down). (**d**) HEK293T cells were co-transfected with plasmids encoding FLAG-tagged Ku70 and/or non-tagged full-length ATG5 or plasmid encoding 1–192 a.a. of ATG5 proteins. Cells were exposed to either Etoposide or DMSO as a vehicle after 24 h post-transfection and IPs were performed using Flag beads. Anti-ATG5, anti-Ku70, anti-Ku80 and anti-FLAG antibodies were used for immunoblotting. Input, total cell extract. ACTB, anti-β-Actin was used as a loading control.

Previous studies addressed the importance of free ATG5 (not-conjugated with ATG12) for its autophagy-independent role^20^. For this reason, we only overexpressed non-tagged human ATG5 (hATG5) in HEK293T cells. We observed that ATG5 overexpression resulted in a Co-IP only with endogenous Ku70 but not with Ku80 (Fig. 2a). Indeed, ATG5-Ku70 interaction was further stimulated by etoposide exposure (Fig. 2a). Moreover, we confirmed this interaction with endogenous Co-IP experiments in HEK293T cells. Our results revealed that endogenous ATG5 only interacted with Ku70 but not with Ku80 and the interaction of endogenous ATG5 and Ku70 proteins was further enhanced in HEK293T cells during genotoxic stress (Fig. 2b). Moreover, we could validate that Ku70 and ATG5 interacted directly according to in vitro binding assays using recombinant proteins (GST-pulldown) (Fig. 2c). We would like to understand whether this interaction requires any specific site of ATG5 protein. To further establish this nature of the physical interaction, we performed Co-IPs with overexpressing Flag-tagged full length or truncated ATG5 (plasmids encoding 1–192 a.a. of the human ATG5, lack of ATG5 C-terminal domain) protein. We identified that Ku70 and ATG5 interaction is more prominent when ATG5 lacks a C-terminal part in HEK293T cells (Fig. 2d).

All these results showed that NHEJ components, including Ku80 were partners of ATG5 during genotoxic stress and Ku70 is a novel ATG5-interacting protein.

### Dynamic nature of ATG5-Ku70 interaction under genotoxic stress conditions

Since our results have shown that Ku70 is a novel ATG5 interactor and this interaction is stimulated by genotoxic stress, we decided to further analyze the dynamics of this interaction.

To further reveal the dynamics of this novel interaction, we performed gel filtration with endogenous proteins in HEK293T cells.

**Figure 3:**
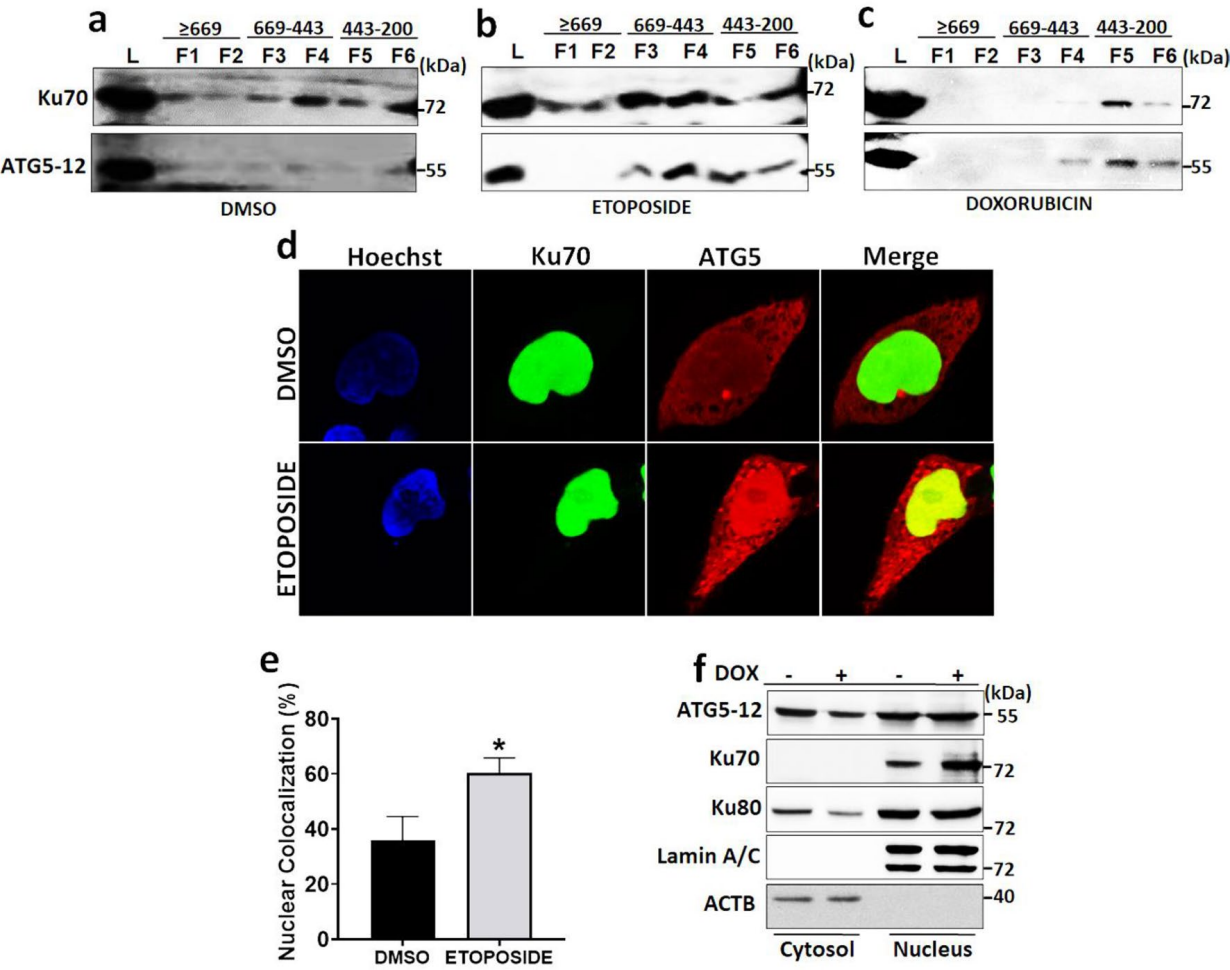
Dynamic nature of ATG5-Ku70 interaction during genotoxic stress. (**a**–**c**) HEK293T cells were exposed to Etoposide (**b**) or Doxorubicin (**c**) treatment for 24 h. DMSO (**a**) was used as a vehicle. Total cell extracts were subjected to gel filtration chromatography. Collected fractions were analyzed by immunoblotting using anti-ATG5 and anti-Ku70 antibodies. Molecular weights of fractions were marked. L, cell lysates before fractionation. (**d**) HEK293T cells were cultured on coverslides and co-transfected with GFP-tagged Ku70 (green) and Cherry-tagged ATG5 (red) constructs. Cells were exposed to Etoposide for 24 h after transfection. DMSO was used as a vehicle. 48 h post-transfection cells were fixed and analyzed under a confocal microscope at 63× magnification. Nuclei were stained with Hoechst (blue). Merge, overlay green and red signals. (**e**) Confocal images were analyzed by counting 100 cells and % of ATG5-Ku70 colocalization in the nucleus was represented in a graph (mean ± S.D. of independent experiments, n = 3, *, *p* < 0.05). (**f**) Cellular fractionation was performed after Doxorubicin treatment. DOX (+), Doxorubicin; (−), DMSO. Nucleus, nuclear fraction; Cytosol, cytosolic fraction; Lysate, the whole-cell lysate was subjected to immunoblotting. Anti-ATG5, anti-Ku70, anti-Ku80, anti-Lamin A/C and anti-β-Actin were used as nuclear and cytosolic fractionation control, respectively.

Protein complexes were separated by using a gel filtration column with a separating range between 5 and 5000 kDa (Supplementary Fig. 4). Previously published data showed that the ATG12-5-16 complex was eluted from the column at 669–800 kDa fractions^19,21^. Gel filtration and all previously described protein–protein interaction assays (Co-IPs and confocal microscopy analyses) established for the first time that, genotoxic stress-induced the interaction of ATG5 and Ku70. Under basal conditions (herein DMSO, as a drug vehicle), ATG5-12 and Ku70 were recovered in the same fractions (Fig. 3a). Surprisingly, following etoposide and doxorubicin treatment, we observed aberrant recruitment of ATG5-12 in the Ku70 containing complexes (Fig. 3b,c). These results indicate a functional and dynamic role of these complexes during genotoxic stress conditions.

Furthermore, a genotoxic stress-dependent increase in ATG5-Ku70 intracellular colocalization was observed under confocal microscopy (Fig. 3d). Ku70 and ATG5 interaction predominantly occurs in the nuclei and significantly increases under genotoxic stress conditions in HEK293T cells (Fig. 3e). Of note, ATG5 interaction with Ku70 did not alter the elimination of this protein, since treatment of cells with autophagy inducer Torin 1, mTOR inhibition, did not result in its degradation (Supplementary Fig. 5a). To further validate nuclear colocalization, we performed cellular fractionation. Under the basal condition, ATG5 was found predominantly in the cytosolic fraction, yet Ku70 and Ku80 proteins were more prominent in the nuclear fraction. Following doxorubicin exposure, ATG5 was translocated to nuclei in HEK293T cells (Fig. 3f). Migration of the Ku70 to nuclei upon genotoxic stress induced NHEJ mechanism was known. Here we determined that ATG5 also translocates to the nuclei regardless of the type of genotoxic stress (e.g., etoposide or doxorubicin) (Fig. 3e,f). We also performed Co-IP by using doxorubicin treated HEK293T fractions and checked the interaction between Ku70 and ATG5. Our data revealed that not only ATG5 translocated to the nuclei, but also interacted with Ku70 upon genotoxic stress (Supplementary Fig. 5b). Therefore, genotoxic stress led to the recruitment of ATG5 and facilitate the interaction with Ku70 in the nucleus.

### ATG5-Ku70 interaction is required for effective DNA damage sensing and repair

Ku70 is a well-known player in the recognition of the DSB damages on DNA. To investigate the functional role of ATG5-Ku70 interaction, we first performed subcellular fractionation experiments and analyzed the abundance of proteins in cytoplasmic versus the nuclear fraction of the cells. In line with co-immunoprecipitation and confocal analyses, a notable fraction of ATG5 was recruited into the nucleus following genotoxic stress (Fig. 3d–f).

**Figure 4:**
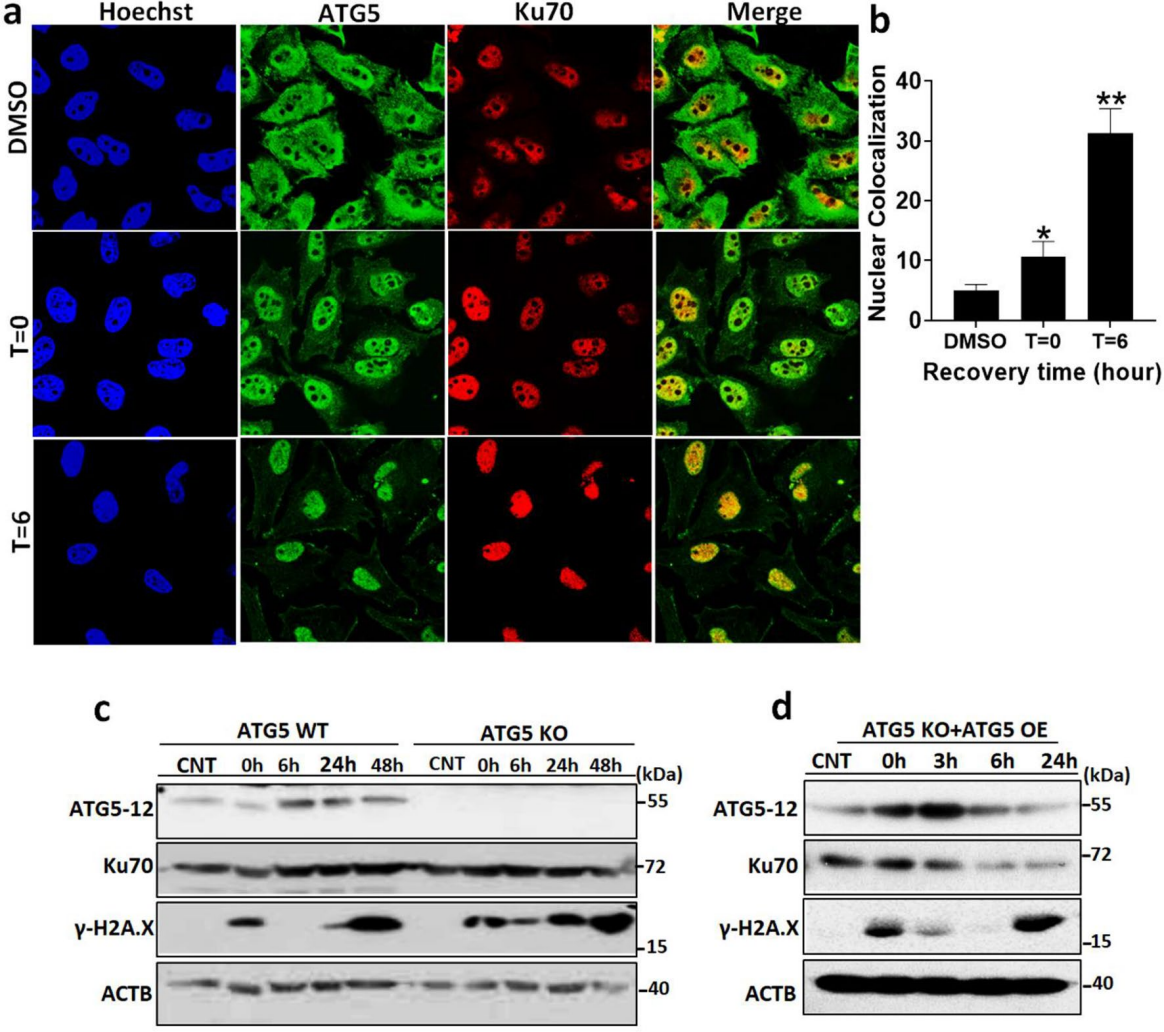
Role of ATG5-Ku70 interaction in DNA damage sensing and repair. (**a**) HeLa cells were cultured on coverslides and exposed to Etoposide for 1 h. DMSO was used as a vehicle. Etoposide was washed out after 1 h and cells were cultured for along 6 h to recover. t = 0, 1 h etoposide treated cells. t = 6, 6 h recovered cells. Cells were fixed and indirect immunofluorescent analysis was utilized. Alexa-488 (green) and Alexa-568 (red) were used as secondary antibodies against rabbit anti-ATG5 and mouse anti-Ku70, respectively. Cells were visualized under a confocal microscope at 63× magnification. Nuclei were stained with Hoechst (blue). Merge, overlay green and red signals. (**b**) Colocalization coefficients of 50 cells were calculated from confocal images and data represented as a graph (mean ± S.D. of independent experiments, n = 3, *, *p* < 0.05). (**c**) HeLa WT and *ATG5* KO cells were treated with Etoposide for 1 h. After treatment etoposide was washed out and cells remained in the culture for along 6 h, 24 h and 48 h for recovery. CNT, DMSO treated cells as a vehicle. Then proteins were collected and analyzed by immunoblotting using anti-ATG5, anti-Ku70, and anti-γH2A.X. Anti-β-Actin was used as a loading control. (**d**) A rescue experiment was performed. HeLa *ATG5* KO cells were cultured and transfected with ATG5 expressing plasmid. 48 h post-transfection cells were treated with etoposide for 1 h. After treatment etoposide was washed out and cells remained in the culture for along 6 h, 24 h and 48 h for recovery. CNT, DMSO treated cells as a vehicle. Then cells were collected and analyzed by immunoblotting using anti-ATG5, anti-Ku70, and anti-γH2A.X. Anti-β-Actin was used as a loading control.

To further assess the importance of the interaction for DNA damage sensing and repair, we designed recovery experiments upon genotoxic stress. Upon DSBs, phosphorylated H2A.X, γ-H2A.X, accumulates on the DNA lesion and leads to the recognition of DSBs^22^. Following etoposide exposure, etoposide was washed out and cells were left for recovery for 6–48 h to monitor repair capacity. To support our previous findings in HEK293T cells, confocal microscopy analysis demonstrated that a notable proportion of ATG5 was recruited to the nucleus upon DNA damage in HeLa cells (Fig. 4a,b). Strikingly, the abundance of ATG5 protein in the nucleus is more prominent during recovery (T = 6, time point) compared to the active drug-exposed condition (T = 0, time point) (Fig. 4a,b). In line with this, DSBs damage occurs following etoposide exposure. γ-H2A.X accumulated following genotoxic stress in HeLa cells (T = 0, time point). After 6 h post-damage, γ-H2A.X accumulation diminished in HeLa (*ATG5* WT HeLa) cells whereas not in autophagy attenuated cells (Fig. 4c).

Although, ATG5 KO HeLa cells accumulate more γ-H2A.X at 24 and 48 h post-damage compared to WT HeLa cells. WT cells were still found γ-H2A.X positive at 48 h post-damage which may indicate the error-prone repair that may eventually causing the arrest and leading the genomic instability.

Next, to further investigate the interaction of ATG with the DNA damage response mechanism, we knocked out *ATG5* in HeLa cells. We first performed a T7 endonuclease I assay to validate the CRISPR-Cas9 editing capacity in our cells. PCR amplified *ATG5* regions were purified and treated with T7E1 and visualized by a UV transilluminator. Editing was successfully validated in our knockout cells which showed clear T7E1 activity compared to *ATG5* WT counterpart (Supplementary Fig. 6a). Moreover, to further validate that these cells exert autophagic attenuation, we performed functional autophagy assays. ATG5 protein and LC3 lipidation, which is a marker of autophagic activity, were totally abolished and *ATG5* null cells accumulated a high amount of p62, an autophagic cargo receptor, upon CRISPR. Moreover, *ATG5* null cells did not respond to starvation, yet starvation (EBSS, 4 h; well-established autophagy inducer) induced autophagy in *ATG5* WT cells as expected (Supplementary Fig. 6b). Indirect immunofluorescent analyses were also showed high p62 accumulation and attenuation of LC3-dot formation (Lapidated LC3 forms dots on autophagosomes) in *ATG5* null HeLa cells in comparison with control (Supplementary Fig. 6c–e). All these data validate that CRISPR-Cas9 was successfully altered the *ATG5* level and caused a severe autophagic attenuation in HeLa cells. Interestingly, *ATG5* null HeLa cells (*ATG5* KO HeLa) manifested a disability to repair DNA damage upon genotoxic stress. At 6 h post-recovery, *ATG5* KO cells still bare γ-H2A.X positive lesion showing unrepaired DNA damage in *ATG5* KO HeLa cells (Fig. 4c).

To validate the direct effect of ATG5-Ku70 interaction in our context, we performed rescue experiments by overexpressing *ATG5* in *ATG5* KO HeLa cells. Strikingly, recovery of *ATG5* restored the repair disability in KO HeLa cells (Fig. 4d).

To further validate the repair capacity, we monitored cell growth following genotoxic stress. Our data demonstrated that there is no significant cell number difference in both *ATG5* KO and control HeLa cells during genotoxic stress (0 h time point) and early time points of recovery (Supplementary Fig. 7a). However, after 6 h post-damage, WT HeLa cells showed greater proliferative capacity as compared to *ATG5* KO HeLa cells (Supplementary Fig. 7a). This data was correlated with the gradually increased γ-H2A.X level at 24 and 48 h post-damage which *ATG5* KO HeLa cells accumulate more γ-H2A.X at 24 and 48 h post-damage compared to WT HeLa cells (Fig. 4c). To understand whether accumulated γ-H2A.X caused decreased proliferation or lead to cell death, we checked apoptosis markers under this condition. Activation and cleavage of both PARP and caspase-3 are considered as markers of apoptosis. None of those were activated upon damage and following recovery (Supplementary Fig. 7b). Hence, our data suggest that accumulated γ-H2A.X following the loss of *ATG5* may lead to the proliferation arrest rather than facilitating cell death.

All these data suggest that ATG5-Ku70 interaction is necessary for the repair capacity of the cells following genotoxic stress.

## Discussion

In the present study, we discovered that: 1, NHEJ complex components including Ku70, Ku80 and DNA-PKcs interact with ATG5; 2, direct protein–protein interactions exist between autophagy protein ATG5 and NHEJ complex components Ku70 and are enhanced upon genotoxic stress condition, 3, for the first time we showed that NHEJ component Ku70 in the same complex with ATG5-12 and it is dynamically enhanced upon genotoxic stress; 4, ATG5 nuclear translocation-induced by genotoxic stress and its interaction with Ku70 predominantly occurs in the nuclei; 5, ATG5-Ku70 interaction in the nucleus is more significant during recovery from the DNA-damage was observed; 6, loss of ATG5 was abolished the repair capacity of cells and enable proliferation following recovery.

Utilizing Tri-SILAC-LC-MS/MS, co-immunoprecipitation, confocal microscopy and gel filtration analyses, we demonstrated that ATG5 interacted with NHEJ components including DNA-PKcs, Ku70 and Ku80. A novel and direct interaction with endogenous ATG5 was validated with Ku70 in the nucleus under basal conditions. Our data revealed that these interactions were highly dynamic and enhanced following genotoxic stress by DNA damage-causing agents.

The protective role of autophagy upon genotoxic stress was previously described^17^. Several studies indicated that ATG5 exerts an alternative function during genotoxic stress independently from its role in autophagy^20,23–25^. Nuclear ATG5 was shown to interact with Survivin and blocked its association with Aurora B, a chromosomal passenger complex protein (CPC) regulating mitosis^20^. In another study, nuclear ATG5 has also been associated with increased drug resistance and microsatellite instability-high (MSI-H) phenotype in colorectal cancer^24^.

In our system, nuclear translocation of ATG5 was increased upon DNA damage and it was more prominent during repair after post-damage. Although several studies reported the nuclear role of ATG5, the role of nuclear ATG5 during repair was not reported.

The role of autophagy proteins in DDR and repair mechanisms has been studied previously^26,27^. Although some autophagy-related proteins have been addressed in the NHEJ mechanisms, no study documented the involvement of ATG5 in NHEJ. Ionizing-radiation (IR)-induced damage caused by the accumulation of UVRAG, autophagy-related protein, facilitates the activation of DNA-PKcs by monitoring the recruitment of DNA-PKcs for the recognition of DSBs^27^. Of note, Loss of UVRAG or attenuation of autophagic activity resulted in impaired repair^27^.

Therefore, in addition to the involvement of individual autophagy-related proteins in the orchestration of DNA repair, the autophagy capacity of cells may be important. Cellular clearance of NHEJ complex proteins may be regulated by two major cellular catabolic processes, UPS and autophagy^28^. Rather than engulfment of cargo by autophagosomes in autophagy, UPS, involves the poly-ubiquitination of target proteins and their degradation in the proteasomes^29^.

Although, they were shown to act individually on different targets, in some contexts, they were co-regulated^30^. Increased autophagic activity following mTOR-inhibition has been associated with reduced UPS and led to the accumulation of Ku80 and XRCC4 in IR-induced DNA damage in hematopoietic stem cells (HSCs)^31^. In another study, DDR protein, checkpoint kinase 1 (Chk1) which plays a critical role in the decision between Homologous repair (HR) and NHEJ was found to be degraded by CMA. Of note, another autophagic degradation system called chaperone-mediated autophagy (CMA) was shown to be enhanced upon inhibition of autophagy^32^. SQSTM1/p62 is a well-known autophagy receptor protein that is degraded upon autophagic activity. p62 was reported to be accumulated on DNA lesions upon genotoxic stress.

Following IR-induced damage, p62 was shown to interact with Filamin A following nuclear translocation. This interaction has facilitated the degradation of both Filamin A and RAD51 by proteasomes into the nucleus which favors NHEJ over HR^33^.

HR is another major repair mechanism responsible for the repair of DSBs^13^. Loss of autophagic activity was reported to cause a shift between these two major DSBs repair mechanisms. IR-induced DNA damage activated autophagy which in turn degrades USP14, deubiquitinase of Ku70. Inhibition of IR-induced autophagy caused accumulation of USP14 on DSBs that enables recruitment of NHEJ components on IR-induced foci. Autophagy deficient cells were identified as insufficient to recruit NHEJ components on DSBs and accumulate more IR-induced foci which were associated with diminished repair capacity^34^.

Deregulation of DNA-PKcs activity or disintegration of Ku70/Ku80 heterodimer may cause severe defects in NHEJ and lead to unrepairable genomic instability. For instance, levels of NHEJ complex components including DNA-PKcs, Ku70 and Ku80, were reported to be upregulated in several cancer cells following radio-and chemotherapy which were hampered the efficacy of the anti-cancer therapy^35^. Thereof, targeting NHEJ may serve a key role to sensitize cancer cells against therapeutics. Anti-cancer drugs were generally designed to target rapidly dividing cancer cells by introducing DNA damage. Unrepaired damages were found to be associated with mitotic catastrophe (MC) and subsequent cell death to avoid genomic instability^36^. Previous studies reported that intact autophagic activity is important for cell death following mitotic catastrophe^20,36,37^. Anti-cancer drug-induced MC was reduced in autophagy incompetent cells. Of note, in some cases, MC was found to be induced upon autophagic activation^37^. In contrast, nuclear ATG5-induced mitotic catastrophe was not found to be linked with elevated cell death^20^.

Our data suggested that cells are more sensitive to anti-cancer drugs when autophagy is intact during recovery. Strikingly, we discovered that loss of ATG5 resulted in attenuation of recovery following post-damage.

Altogether, our study revealed an important interaction between autophagy machinery and NHEJ repair during genotoxic stress. There has been no published data showing that ATG5 is involved in NHEJ. We discovered for the first time that ATG5-Ku70 interaction is required for the efficacy of repair following genotoxic stress. Hence, we proposed a new role of autophagy protein ATG5 in DNA-damage repair following genotoxic stress.

## Materials and methods

### Plasmid constructs

Nucleotides covering 1 to 192 a.a. of human ATG5 cDNA insert in the pcDNA3 construct cut and ligated into a pCMV-3Tag-6 Flag expression vector. For GST-tagged protein production, human Ku70 was cloned into the BamHI and EcoRI sites of bacterial expression vector pGEX-4 T-1-3xFLAG (Addgene, #129570) followed by digestion with BamHI and EcoRI from pEGFP-C1-FLAG-Ku70 (Addgene, #46957) plasmid. hATG5 vector was kindly gifted from Noboru Mizushima. Flag and GFP tagged human Ku70 (Addgene, #46957) and Ku80 (Addgene, #46958), pmCherry-ATG5 (Addgene, #13095) plasmid were all provided by Addgene.

### Production and purification of recombinant GST-Ku70 protein

pGEX-4 T-1-3xFLAG-Ku70 was transformed into of protease mutant strain of *E. coli*, BL21 (DE3) and grown at 37 °C until an optical density of 0.3–0.5 was achieved. The GST-fusion protein was induced with 1 mM isopropyl β-d-1 thiogalactopyranoside (IPTG) at 20 °C for 12 h. To observe protein production in cells, bacteria samples were taken and lysis with SDS-PAGE loading dye and loaded on SDS-PAGE. Coomassie staining was used to visualize produced protein in SDS-PAGE gel in comparison to bacteria without IPTG induction. Once induction was verified, the bacterial pellet was resuspended in an adequate amount of lysis buffer (500 mM NaCl, 50 mM Tris–HCl pH:8.0, 2 mM EDTA and, protease and phosphatase inhibitors) containing 1% Sarcosyl. Then, the bacteria solution was sonicated for 4 min (amplitude: 38%, 2 s pulse on, 5 s pulse off), centrifuged at 3000 g, + 4 °C for 1 h. To purify GST-Ku70 from the protein mixture, the supernatant was loaded onto Glutathione Sepharose 4B beads (GE Healthcare, #17-0756-01) in the presence of protease inhibitors overnight at + 4 °C.

To purify protein from the solution, beads were centrifuged, washed and treated with an adequate amount of pull down buffer (20 mM L-Reduced Glutathione (Fluka Analytical, 49750) 50 mM Tris HCI pH: 8.0, 5% glycerol, final pH:8.0) for 15 min while rotating at RT. After the incubation period, beads were centrifuged, and supernatant stored at − 80 °C for further studies.

### GST pulldown assay

For pulldown assay 25 μl of Glutathione-Sepharose 48 beads (GE Healthcare, Cat no: 17-0756-01) in Tris–HCl (20 mM, pH 7.5), protease and phosphatase inhibitors were initially incubated (o/n at 4 °C) with 20 μl of GST-tagged Ku70 recombinant protein that we previously produced (Supplementary Fig. 3). Following six washes in the same buffer, beads were incubated for 8 h at 4 °C with 20 ng of His-tagged ATG5 recombinant protein (Novus, H00009474-P01). Samples were analyzed by immunoblotting as described.

### Cell culture and chemicals

HEK293T and HeLa WT and ATG5 KO cells were cultured in full DMEM medium, which is DMEM (Sigma-D5671) supplemented with 10% (v/v) fetal bovine serum (FBS), L-glutamine and 100 U/mL penicillin/streptomycin, at 37 °C in a humidified 5% CO_2_ incubator. The calcium phosphate salt precipitation method was used as a transfection method for both cell lines^38^. For chemical induction of autophagy, cells were incubated for 3 h in media containing Torin 1 (250 nm, Tocris, catalog no. 4247) that were dissolved in DMSO (Sigma, D2650). Starvation (EBSS, 4 h treatment) was also used to induce autophagy in cells. Doxorubicin (Sigma, D1515), Etoposide (Sigma, E1383) and Cisplatin (Sigma, P4394) that were dissolved in DMSO (Sigma, D2650) were used in DNA damage induction.

### Establishment of *ATG5* KO HeLa cells

HeLa *ATG5* KO cells were generated in our laboratory by using the CRISPR/Cas9 system. gRNA sequences for targeting *ATG5* were designed by using sgRNA Designer: CRISPRko Broad institute online tool. Complementary oligonucleotides were purchased including restriction enzyme BsmBI (Thermo, ER0451) cutting sites. Oligos were annealed and then cloned into lentiCRISPRv2 (Addgene #52961). The calcium phosphate salt precipitation method was used to produce lentiviruses. HEK293T cells were transfected with gRNA-ATG5 containing lentiCRISPRv2 vector with pMD2.G (Addgene #12259) and psPAX2 (Addgene #12260) vectors. After 48 h, the medium was collected, centrifuged, and filtered with a 0.45 µm pore syringe. HeLa cells were transduced with lentiviruses in the presence of 5 µg/ml of polybrene. 48 h later, the selection was carried out by the administration of selection antibiotic puromycin (1 µg/ml) for 5 days. Individual clones were allowed to grow and verified by immunoblotting.

### T7E1 assay

Genomic DNA was extracted from WT and *ATG5* KO cells. The genomic region of ATG5 was amplified by PCR using specific primers. ATG5 Fwd: 5′ ATGACAGATGACAAAGATGTGC 3′ and ATG5 Rev: 5′ATCTGTTGGCTGTGGGATGATA 3′. Amplified DNA was purified by using a PCR clean-up kit (MN, REF 740609.50) and treated with T7E1 (NEB, M0302S) enzyme following the manufacturer’s instructions and visualized following agarose gel electrophoresis.

### Trypan blue exclusion assay

In the recovery experiment, after cells were treated with etoposide for 1 h, the drug was removed, washed twice with 1X PBS and the media was replenished. Cells were trypsinized at different recovery times and subjected to trypan blue. Dead and live cells were counted by light microscopy.

### Immunoblotting

Protein extraction was performed with RIPA buffer (50 mM TRIS–HCl pH 7.4, 150 mM NaCl, 1% NP40, 0.25% Na-deoxycholate) supplemented with complete protease inhibitor cocktail (Roche, 04-693-131-001) and 1 mM phenylmethylsulfonyl fluoride (PMSF; Sigma-Aldrich, P7626). For phosphorylated proteins, protein extraction was performed with RIPA buffer supplemented with a complete protease inhibitor cocktail and phenylmethylsulfonyl fluoride (PMSF; Sigma-Aldrich, P7626) and 100 nM okadaic acid, 1 μM cyclosporine A, 1 mM NaF, 50 mM β-glycerophosphate. Cell extracts were separated by SDS-polyacrylamide gels and transferred to a nitrocellulose membrane. Following blockage in 5% nonfat milk (or 3% BSA for phosphorylated-protein analysis), membranes were incubated in 3% BSA-PBST solutions containing primary antibodies (ab): anti-ATG5 ab (Sigma, A0856, 1:1000), anti-Ku70 ab (Bethyl Laboratories, A302-624A, 1:1000), anti-Ku80 ab (Santa Cruz, sc-5280, 1:1000), anti-FLAG ab (Sigma, F3165, 1:10000), anti-LC3B ab (CST, #2775, 1:1000), anti SQSTM1/p62 ab (BD, 610832, 1:4000), anti-H2AX ab (CST, #2995, 1:1000), anti-γ-H2A.X ab (MerckMillipore, 05-636, 1:1000), and anti β-actin ab (Sigma-Aldrich, A5441, 1:10000). Then, the appropriate secondary mouse or rabbit antibodies coupled to horseradish peroxidase (anti-mouse: Jackson Immunoresearch Laboratories, 115035003; anti-rabbit: Jackson Immunoresearch laboratories, 111035144, 1:10000) were applied and protein bands were revealed with chemiluminescence. The band signals were quantified using ImageJ.

### Immunoprecipitation (IP) tests

For the immunoprecipitation of FLAG-tagged proteins, cells were scraped and lysed using RIPA buffer. An equal amount of protein (1 mg) was incubated with an anti-FLAG M2 affinity gel (Sigma, catalog no. A2220).

Protein G or A-agarose beads (Santa Cruz, sc-2002 and sc-2001, respectively) were coupled overnight with 10 µg/ml of anti-Ku70 or anti-ATG5 antibodies. Normal rabbit or mouse serum (Santa Cruz, sc-2027 and sc-45051, respectively) was used as a negative control. Antibody or serum coupled beads were then incubated overnight with 2 mg lysates and IPs were analyzed by immunoblotting with appropriate antibodies. EtBr (50 μg/ml) was used in the IP to control DNA-dependent and independent protein association.

### Cellular fractionation

Cells were homogenized with isotonic extraction buffer 40 times using a glass Dounce homogenizer and centrifuged at low speed to separate cytosolic fractions. Then nuclei were treated with hypertonic extraction buffer containing (400 mM KCl) and centrifuged to obtain nuclear fraction. Cell extracts were separated by SDS-polyacrylamide gels and transferred to a nitrocellulose membrane. Following blockage in 5% skimmed milk (or 3% BSA for phosphorylated-protein analysis), membranes were incubated in 3% BSA-PBST solutions containing primary antibodies (ab): anti-ATG5 ab (Sigma, A0856, 1:1000), anti-Ku70 ab (Bethyl Laboratories, A302-624A, 1:1000), anti-Ku80 ab (Santa Cruz, sc-5280, 1:1000), anti-H2A.X ab (CST, #2595, 1:1000), and anti β-actin ab (Sigma, A5441, 1:10000). Then, the appropriate secondary mouse or rabbit antibodies coupled to horseradish peroxidase (anti-mouse: Jackson Immunoresearch Laboratories, 115035003; anti-rabbit: Jackson Immunoresearch laboratories, 111035144, 1:10000) were applied and protein bands were revealed with chemiluminescence. Immunoreactive bands were developed on autoradiography films and quantified using ImageJ software.

### Immunofluorescence staining

Cells were fixed with 4% paraformaldehyde and permeabilized in PBS with 0.1% BSA (Sigma, A4503) and 0.1% saponin (Sigma, 84510). Immunostaining was performed using an anti-Ku70 antibody (Santa Cruz, sc-17789), anti-ATG5 ab (Sigma, A0856), and anti-p62 (Abnova, H00008878-M01) and anti-LC3 (Sigma, L8918). As secondary antibodies anti-mouse IgG Alexa Fluor 488 (Invitrogen, catalog no. A11001), anti-rabbit IgG Alexa Fluor 488 (Invitrogen, catalog no. A11008), anti-rabbit IgG Alexa Fluor 568 (Invitrogen, catalog no. A11011) were used. Cells also were co-stained with Hoechst for 10 min. (BD 33,442). Cover slides were mounted and inspected under 63× magnification using a Carl Zeiss LSM 710 confocal microscope (Zeiss, Germany).

### Gel filtration analysis (FPLC)

A SuperoseTM 6 10/300 GL column (separation range 5–5000 kDa) was used (GE Healthcare, catalog no. 17-5172-01) for the separation of proteins. Sigma molecular weight marker sample was used in the calibration of the system (Sigma, catalog no. MWGF-1000). For chromatography analyses, the separation column was connected to the AKTA Prime FPLC system (AKTA FPLC UPC900/P920 System/Frac 900 fraction collector, GE Healthcare). Proteins were separated by using a modification of a previously reported protocol^19,21^. Briefly, to optimize flux, absorbance, and pressure parameters (pressure, 1.5 MPa; flux velocity, 0.5 ml/min; fraction volume, 0.5 ml; loop volume, 500 μl) calibration of the chromatography column was performed using sonicated balancing buffer (1:1, 0.05% glycerol PBS/RIPA buffer). Protein samples extracted from HeLa cells (7 mg of protein) were loaded in a 500 μl volume and collected as 500-μl fractions.

After each sample loading, the column was washed with 36 ml of deionized water (3 × column volume) and re-calibrated with sonicated balancing buffer. Collected fractions were immunoblotted as described.

### SILAC labeling and LC–MS/MS

SILAC-based mass spectrometry analysis, LC–MS/MS and MS data analysis were performed as previously described^19^. Tri-SILAC labeled FLAG-tagged ATG5 expressing HEK293T and HeLa cell extracts were isolated from them and subjected to the analyses.

### Statistical analysis

Statistical analyses were performed using Student’s two-tailed t-test or ANOVA using Graph Pad Prism 8.01 software. Data were presented as means of ± SD of ≥ 3 independent experiments. Values of *p* < 0.05 were considered significant.

### Consent for publication

Authors declare their consent for publication.

## Supplementary Information

Supplementary Information 1.

Supplementary Information 2.

## Data Availability

The datasets used and/or analyzed during the current study are available from the corresponding author on reasonable request.

## Abbreviations

DDR: DNA damage response
NHEJ: Non-homologous end-joining repair system
ROS: Reactive oxygen species
DDR: DNA damage response
SSBs: Single-strand breaks
DSBs: Double-strand breaks
HR: Homologous recombination
DNA-PKcs: DNA-dependent protein kinase catalytic subunit
SILAC: Stable isotope labeling by amino acids in cell culture
Co-IP: Co-immunoprecipitation
IR: Ionizing-radiation
UPS: Ubiquitin-proteosome system
HSCs: Hematopoietic stem cells
Chk1: Checkpoint kinase 1
CMA: Chaperone-mediated autophagy
IAPs: Inhibitor-of-apoptosis proteins
MSI-H: Microsatellite instability-high
MMR-D: DNA mismatch repair deficiency
CPC: Chromosomal passenger complex
MC: Mitotic catastrophe
EtBr: Ethidium bromide
