# Supplementary material for: A novel ATG5 interaction with Ku70 potentiates DNA repair upon genotoxic stress
Source: Sci Rep. 2022 May 17;12:8134. doi: 10.1038/s41598-022-11704-9 (PMC9114114; doi:10.1038/s41598-022-11704-9)

**Supplementary Information**

**A novel ATG5 interaction with Ku70 potentiates DNA repair upon genotoxic stress**

Sinem Demirbag-Sarikaya^1,^*, Yunus Akkoc^2,^*, Sıla Turgut^2^, Secil Erbil-Bilir^1^, Nur Mehpare Kocaturk^1^, Joern Dengjel^3^, Devrim Gozuacik^1,2,4^

**Supplementary Figure Legends**

**Supplementary Figure 1. Tri-SILAC LC-MS/MS analyses for revealing ATG5 proteome.** (**a-c**) Enrichment of Ku70, Ku80 and DNA-PKcs compared with beads alone were represented as a graph, respectively.

**Supplementary Figure 2.** NHEJ components Ku70 and Ku80 are DNA-independent novel interactors. (**a**) HEK293T cells were co-transfected with plasmids encoding FLAG-tagged Ku70 and/or non-tagged full-length ATG5 proteins. (**b**) HEK293T cells were co-transfected with plasmids encoding FLAG-tagged Ku80 and/or non-tagged full-length ATG5 proteins. (**c**) HeLa cells were co-transfected with plasmids encoding FLAG-tagged Ku70 and/or non-tagged full-length ATG5 proteins. (**d**) HeLa cells were co-transfected with plasmids encoding FLAG-tagged Ku80 and/or non-tagged full-length ATG5 proteins. Cells were exposed to Etoposide, Doxorubicin and Cisplatin after 24 h post-transfection. 50 µM Etoposide, 12.5 µg/ml Cisplatin, 1 µm Doxorubicin; 25 µM Etoposide, 1 µg/ml Cisplatin, 100 nm Doxorubicin were used for HEK293T and HeLa cells, respectively. 48 h later, IPs were performed using FLAG beads. EtBr (50 μg/ml) was used to inhibit the effect of the presence of DNA. Anti-ATG5 and anti-FLAG antibodies were used for immunoblotting. Input, total cell extract. Molecular Mass was shown in kilodaltons (kDa).

**Supplementary Figure 3. Production and validation of recombinant GST-Ku70 protein. (a)** Validation of IPTG induction by Coomassie Blue following SDS-PAGE (**-**: no induction, **+**: 1 mM IPTG induction 20°C for 12 h). **(b)** Validation of recombinant GST-Ku70 by Coomassie Blue following SDS-PAGE in the elution (+: 1 mM IPTG induction 20°C for 12 h). (**c**) Immunoblot validation of GST-Ku70 in the elution. HEK293T cell lysates were used as a positive control. (Lanes: 1 and 2 non-induced bacterial lysates, 3: 20 μl GST-Ku70 elute, 4: 10 μl GST-Ku70 elute, 5: 5 μl GST-Ku70 elute, 6-8: HEK293T lysate 100μg total protein)

**Supplementary Figure 4. Gel filtration chromatography analysis.** (**a**) Chromatogram showing peaks of the molecular weight marker mix (Sigma, catalog no. MWGF1000); Ve, elution volume. (**b**) OD595 absorbance confirmation of the peaks.

**Supplementary Figure 5. Ku70 is not an autophagy target and ATG5 and Ku70 interactions are also valid in the nucleus.** Torin 1 was used to induce autophagy (250 nm, 3 h) in HeLa cells. Collected proteins were analyzed by immunoblotting using anti-p62, anti-LC3 and anti-Ku70 antibodies. Anti-β-Actin was used as a loading control. The cell lysate was subjected to Ku70 IP following cellular fractionation using anti-Ku70 coupled agarose beads. Anti-ATG5 was used to control Co-IP.

**Supplementary Figure 6.** **Validation and characterization of *ATG5* KO HeLa cells.** (**a**) T7E1 assay was utilized both on WT and *ATG5* KO HeLa cells. (**b**) Autophagy is characterized by LC3-shift and p62 accumulation assays by immunoblotting. Loss of ATG5 protein was also evaluated. 4h EBSS treatment was used to starve cells. (**c**) Confocal indirect immunofluorescent analysis was performed by using anti-LC3 and anti-p62 antibodies. As a secondary Alexa-488 (green) and Alexa-568 (red) antibodies were used. Nuclei stained with Hoechst (blue) to stain both WT and *ATG5* KO HeLa cells. (**d**) LC3 dot formation was evaluated.

Each dot in 150 cells was counted and an average number of dots/per cell was validated. LC3 dot positivity includes the cells which are above the threshold. 100 cells were analyzed and data were represented as a graph. (**e**) P62 aggregate/cell was evaluated under a microscope and data was represented as a graph.

**Supplementary Figure 7. Loss of ATG5-Ku70 interaction enables recovery of genotoxic stress.** (**a**) HeLa WT and *ATG5* KO cells were treated with Etoposide for 1 h. After treatment, Etoposide was washed out and cells remained in the culture for along 6 h, 24 h and 48 h for recovery. CNT, DMSO treated cells as a vehicle. Then cell viability was assessed by trypan blue exclusion assay (mean ± S.D. of independent experiments, n = 3, n.s. by two-way Anova). (**b**) HeLa WT cells were treated with Etoposide for 1 h. After treatment, Etoposide was washed out and cells remained in the culture for along 6 h, 24 h and 48 h for recovery. CNT, DMSO treated cells as a vehicle. Then cell lysate was well analyzed by using apoptosis markers including PARP and caspase 3. (Cleaved-PARP(c-PARP) and cleaved-Caspase-3 (c-C3)). ACTB was used as a loading control.

**Supplementary Fig. S1**


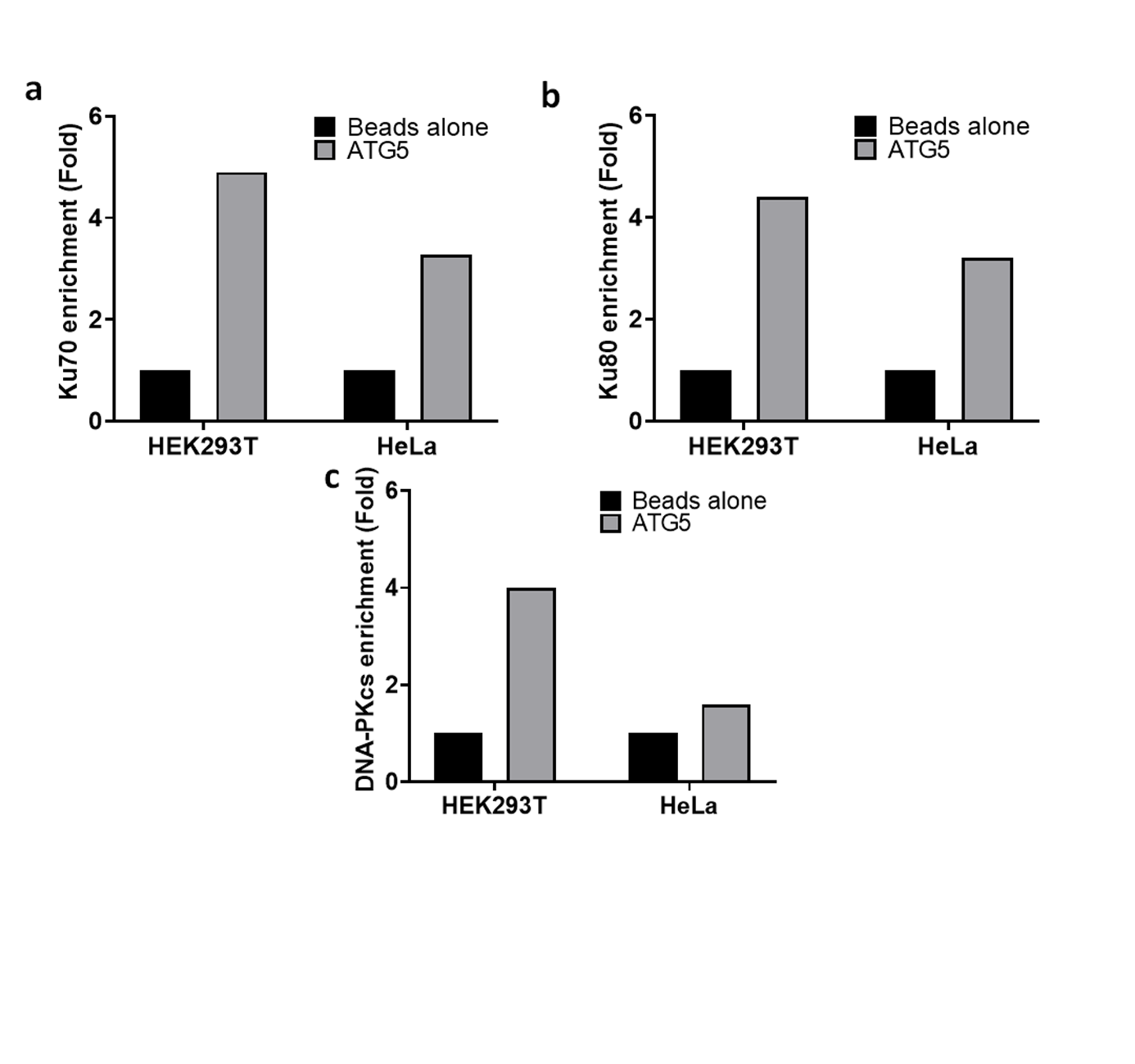


**Supplementary Fig. S2**


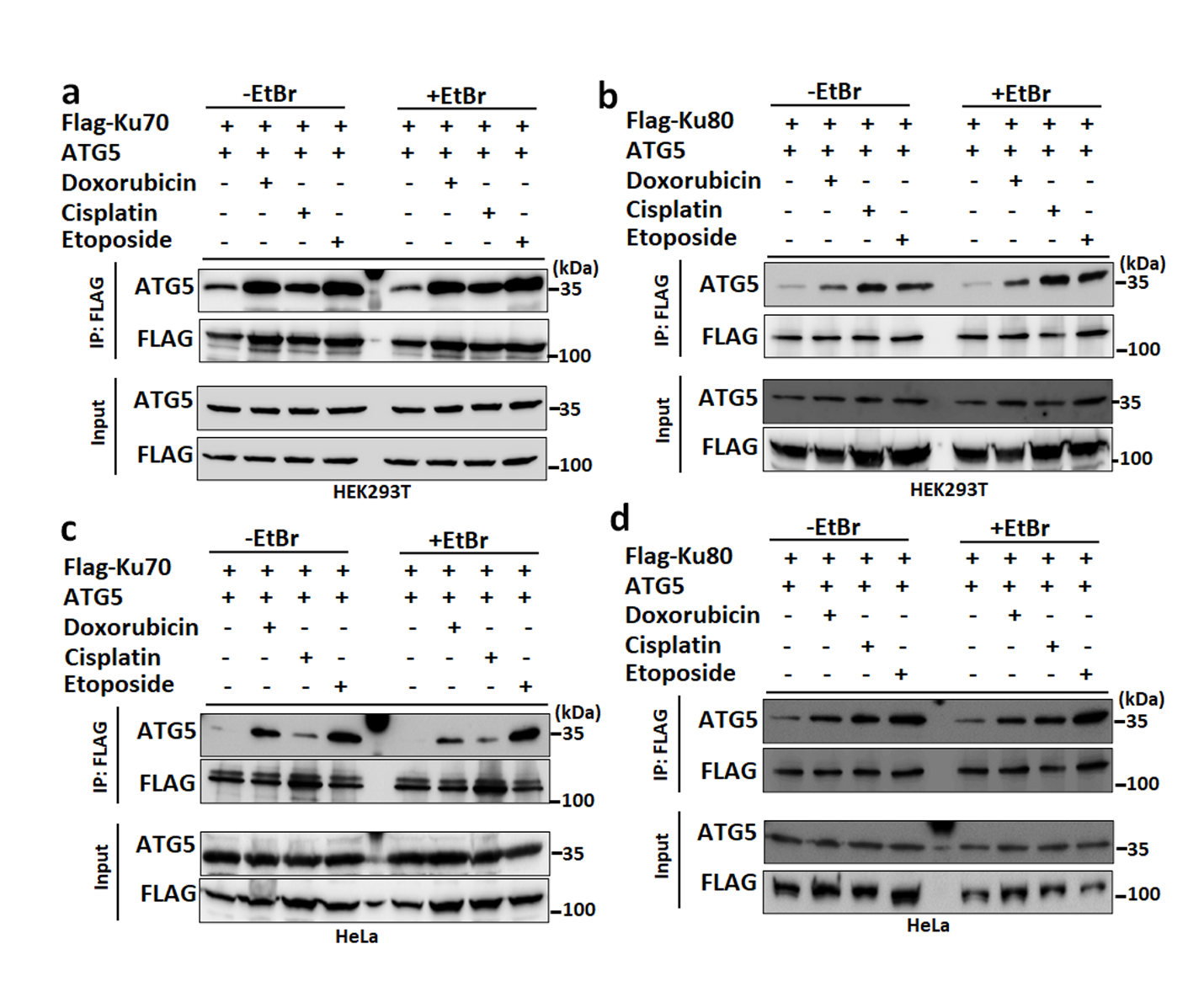


**Supplementary Fig. S3**


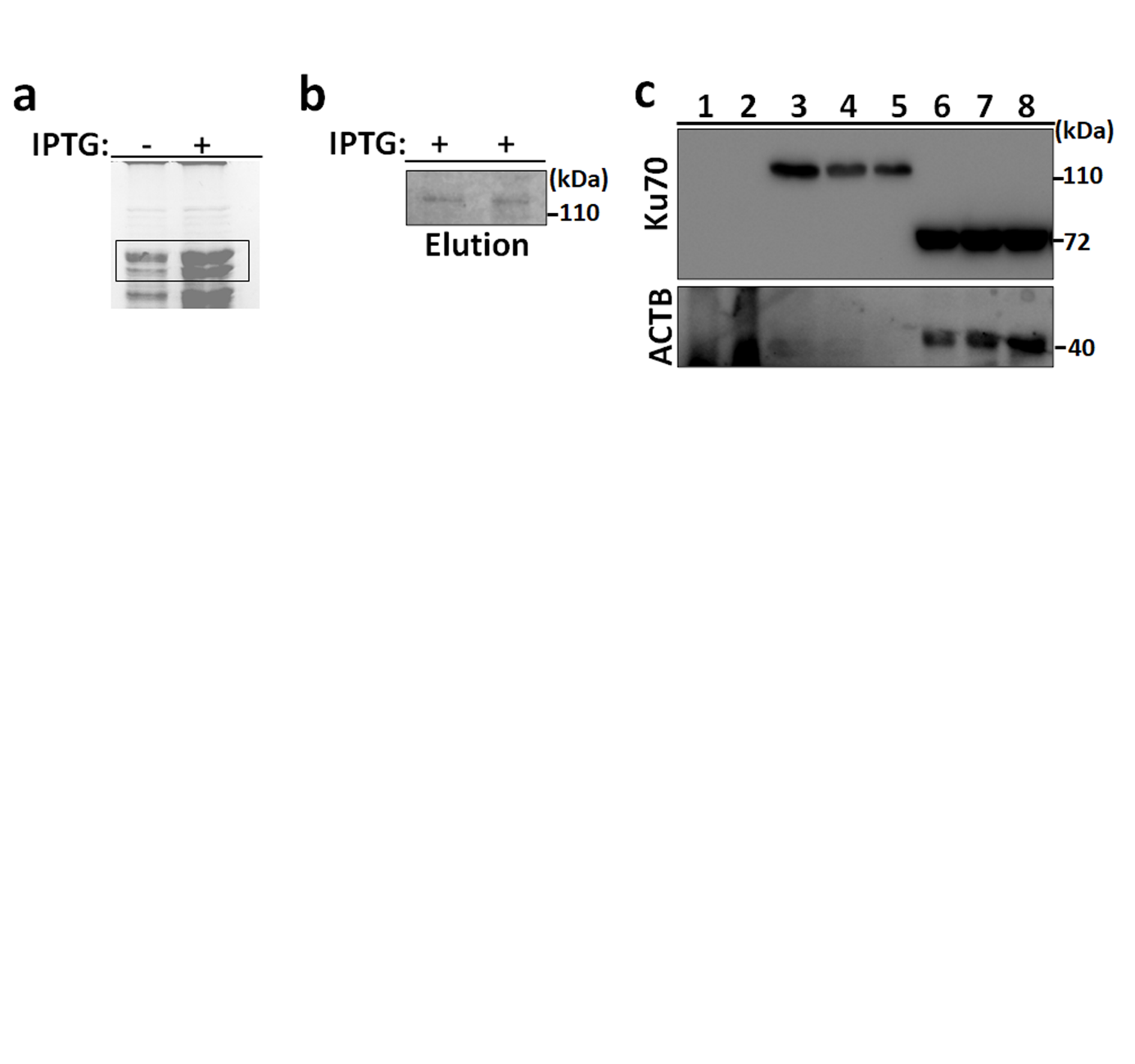


**Supplementary Fig. S4**


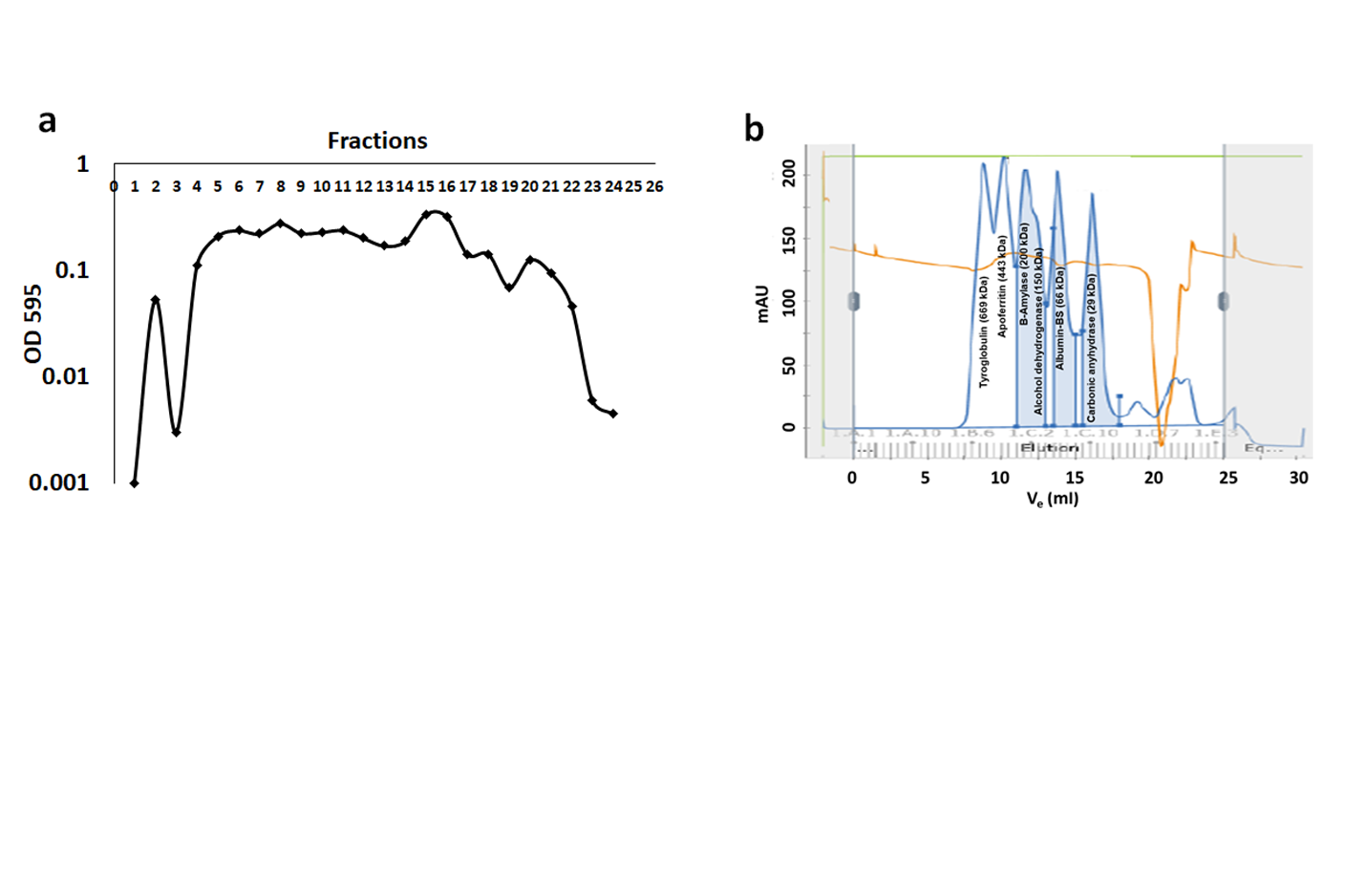


**Supplementary Fig. S5**


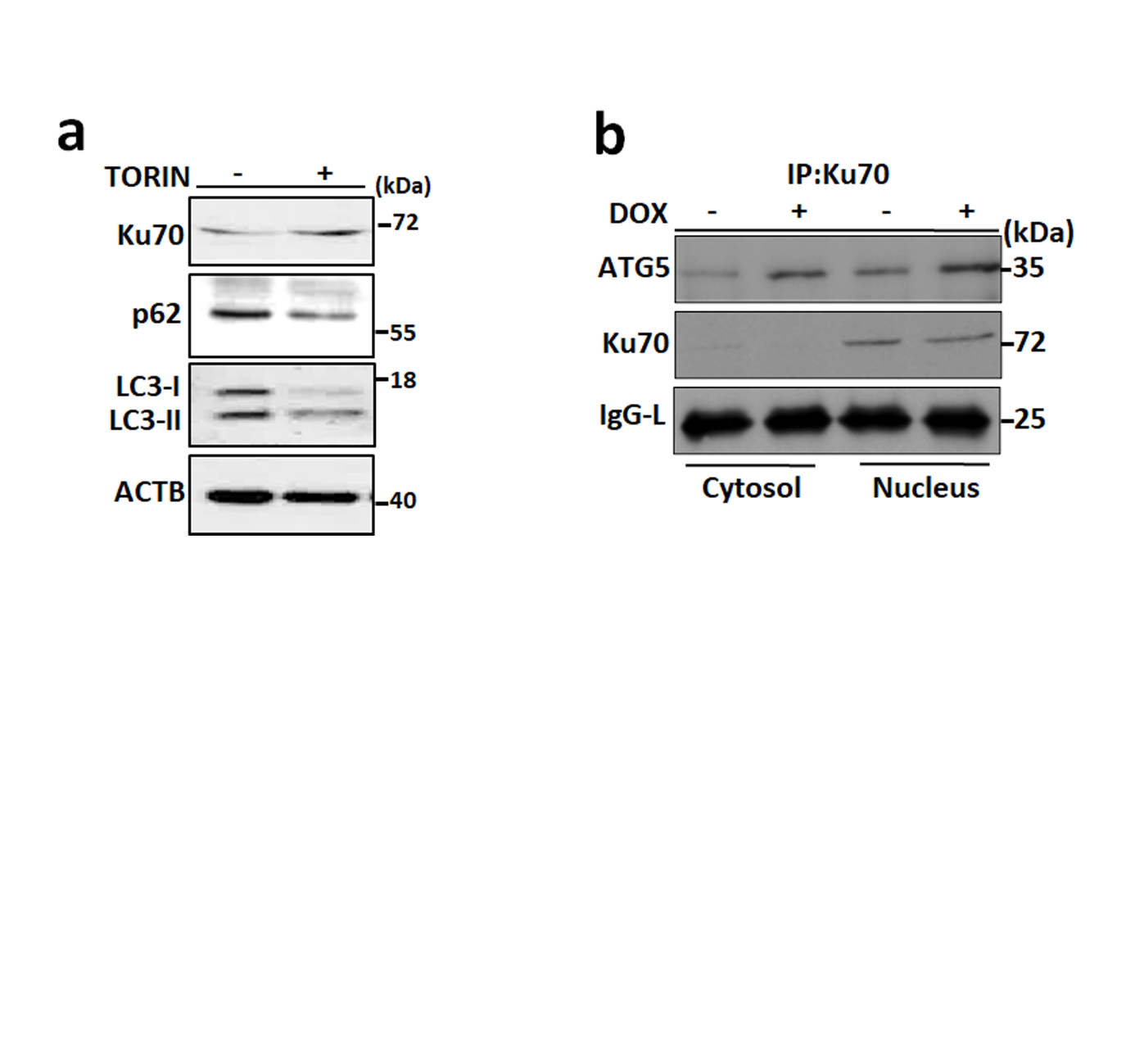


**Supplementary Fig. S6**


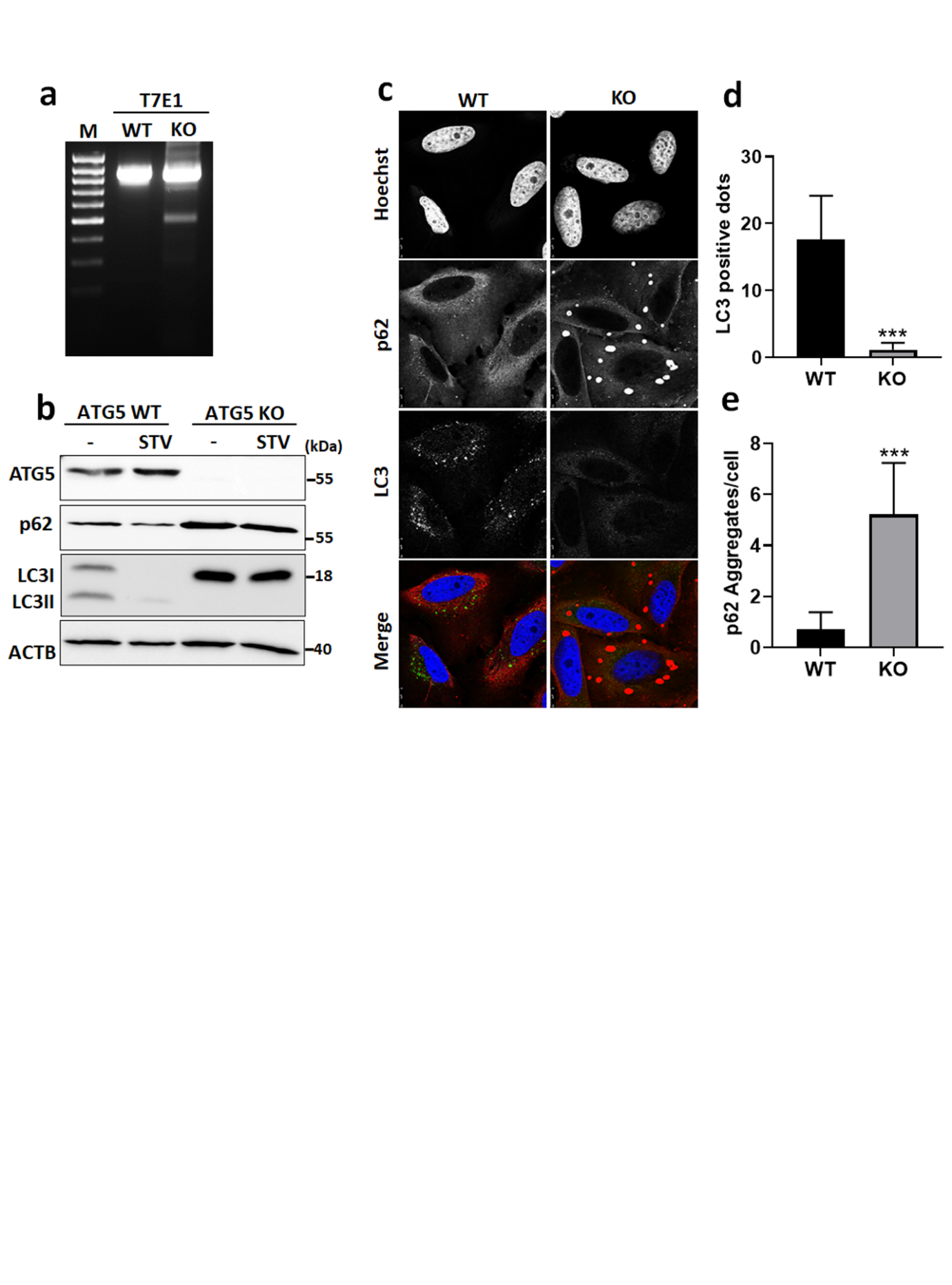


**Supplementary Fig. S7**


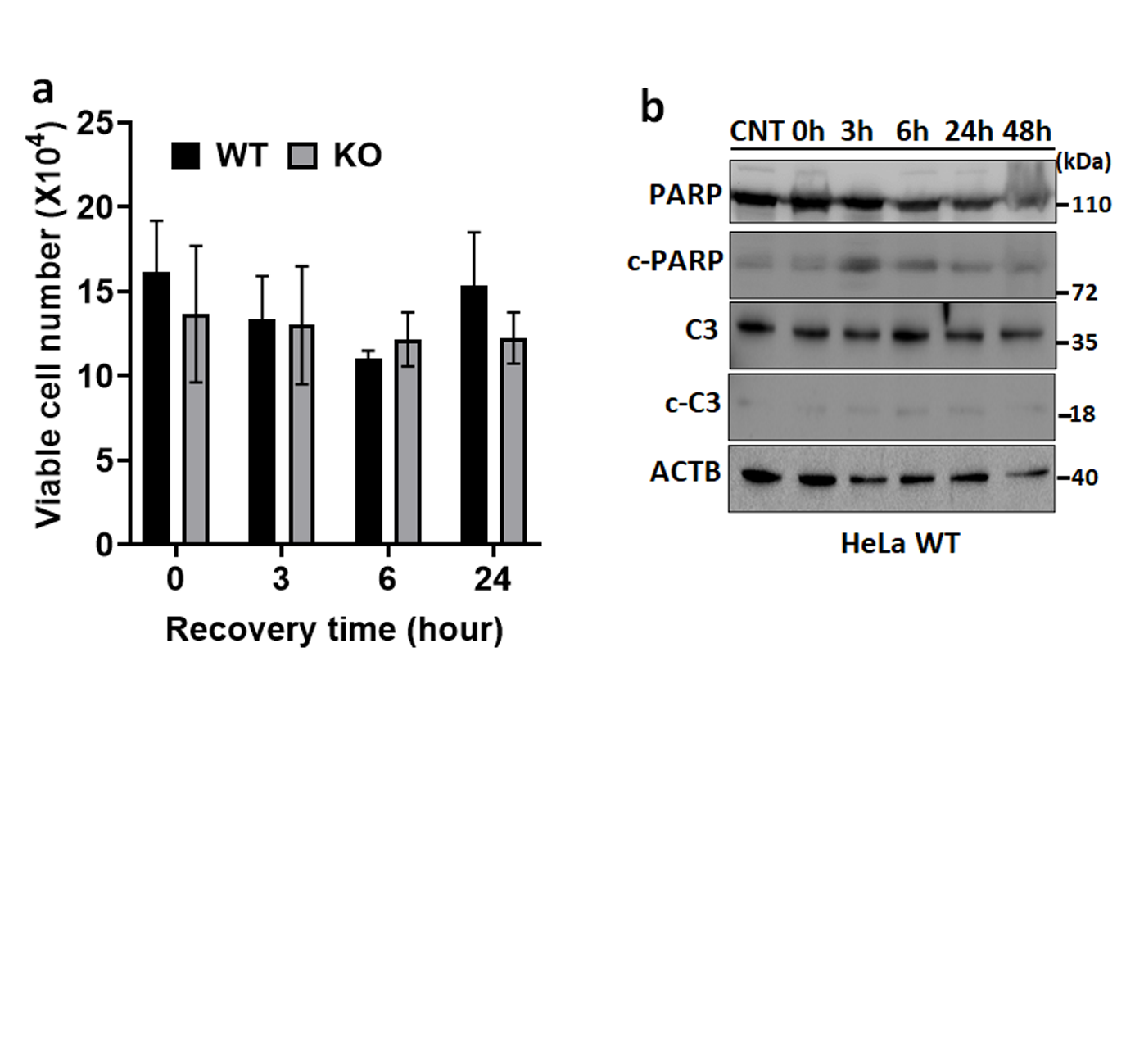

Supplement: Supplementary file 1 — Supplementary Information 1. [file 41598_2022_11704_MOESM1_ESM.docx]
