# Supplementary material for: A novel ATG5 interaction with Ku70 potentiates DNA repair upon genotoxic stress
Source: Sci Rep. 2022 May 17;12:8134. doi: 10.1038/s41598-022-11704-9 (PMC9114114; doi:10.1038/s41598-022-11704-9)

Figure 1a

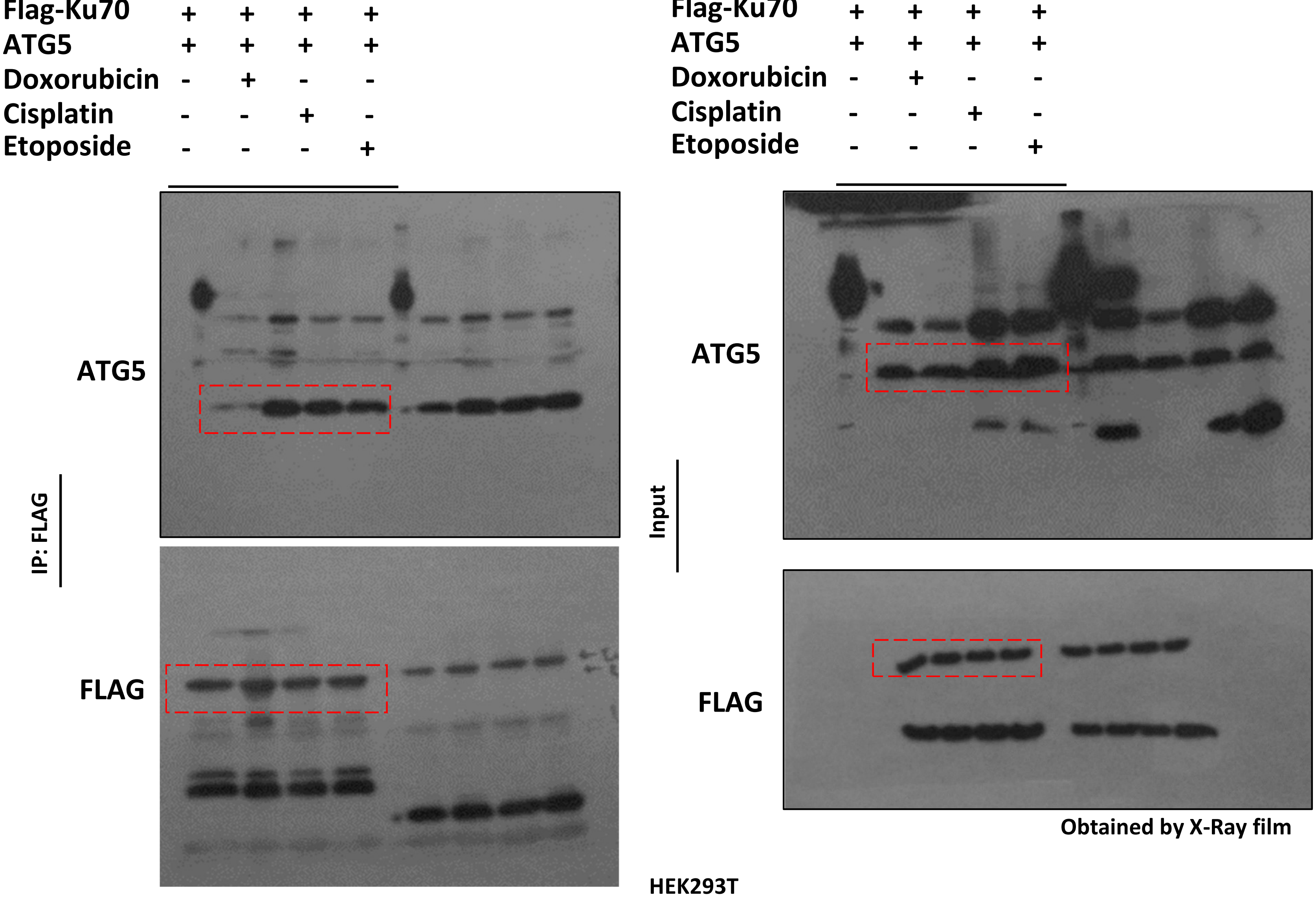
Flag-Ku70 + + + +
ATG5 + + + +
Doxorubicin - + - -
Cisplatin - - + -
Etoposide - - - +
ATG5
FLAG
IP: FLAG
Flag-Ku70 + + + +
ATG5 + + + +
Doxorubicin - + - -
Cisplatin - - + -
Etoposide - - - +
ATG5
FLAG
Input
Obtained by X-Ray film
HEK293T

Figure 1b

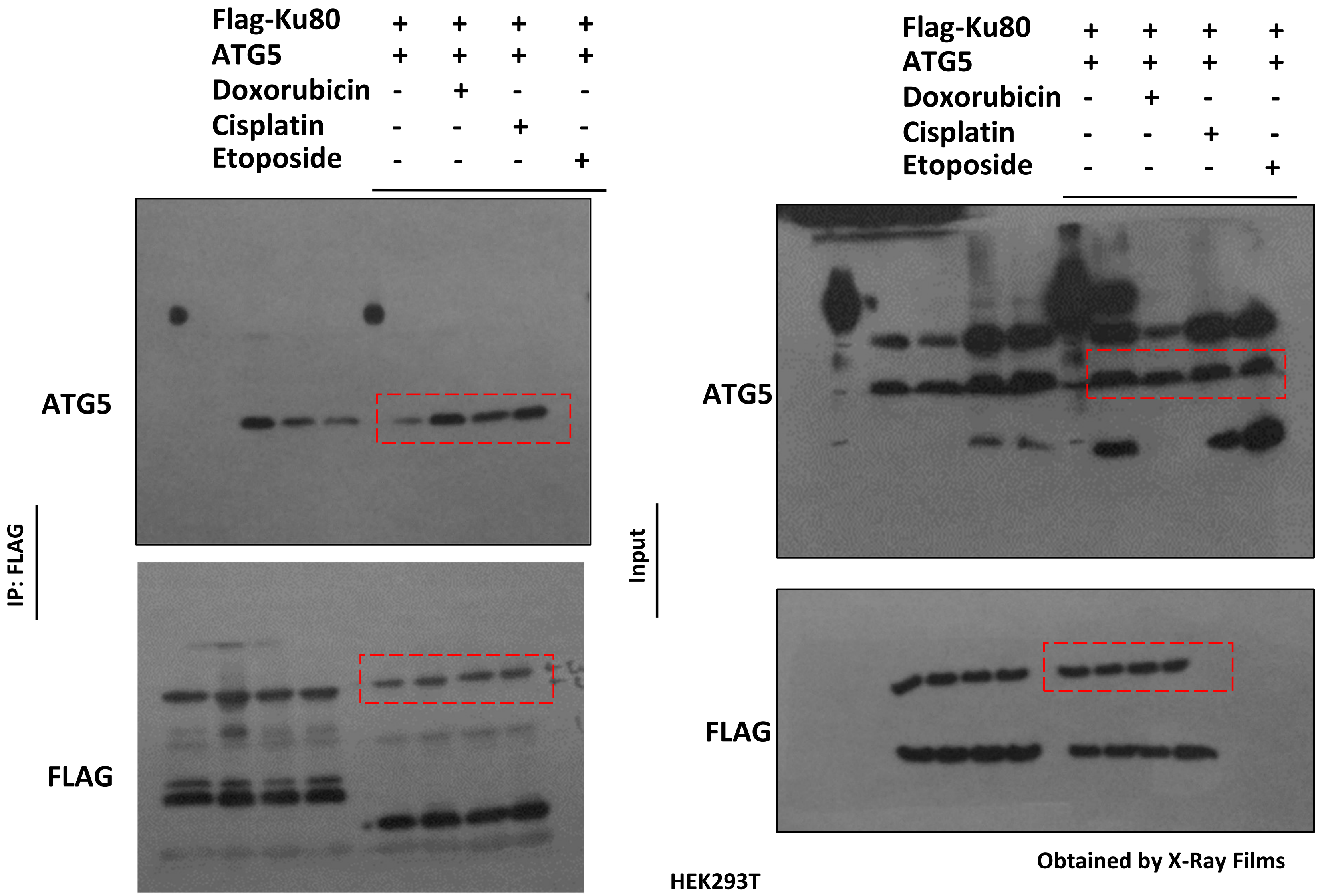

Flag-Ku80 + + + +
ATG5 + + + +
Doxorubicin - + - -
Cisplatin - - + -
Etoposide - - - +
ATG5
FLAG
IP: FLAG
Flag-Ku80 + + + +
ATG5 + + + +
Doxorubicin - + - -
Cisplatin - - + -
Etoposide - - - +
ATG5
FLAG
Input
HEK293T
Obtained by X-Ray Films

Figure 1c

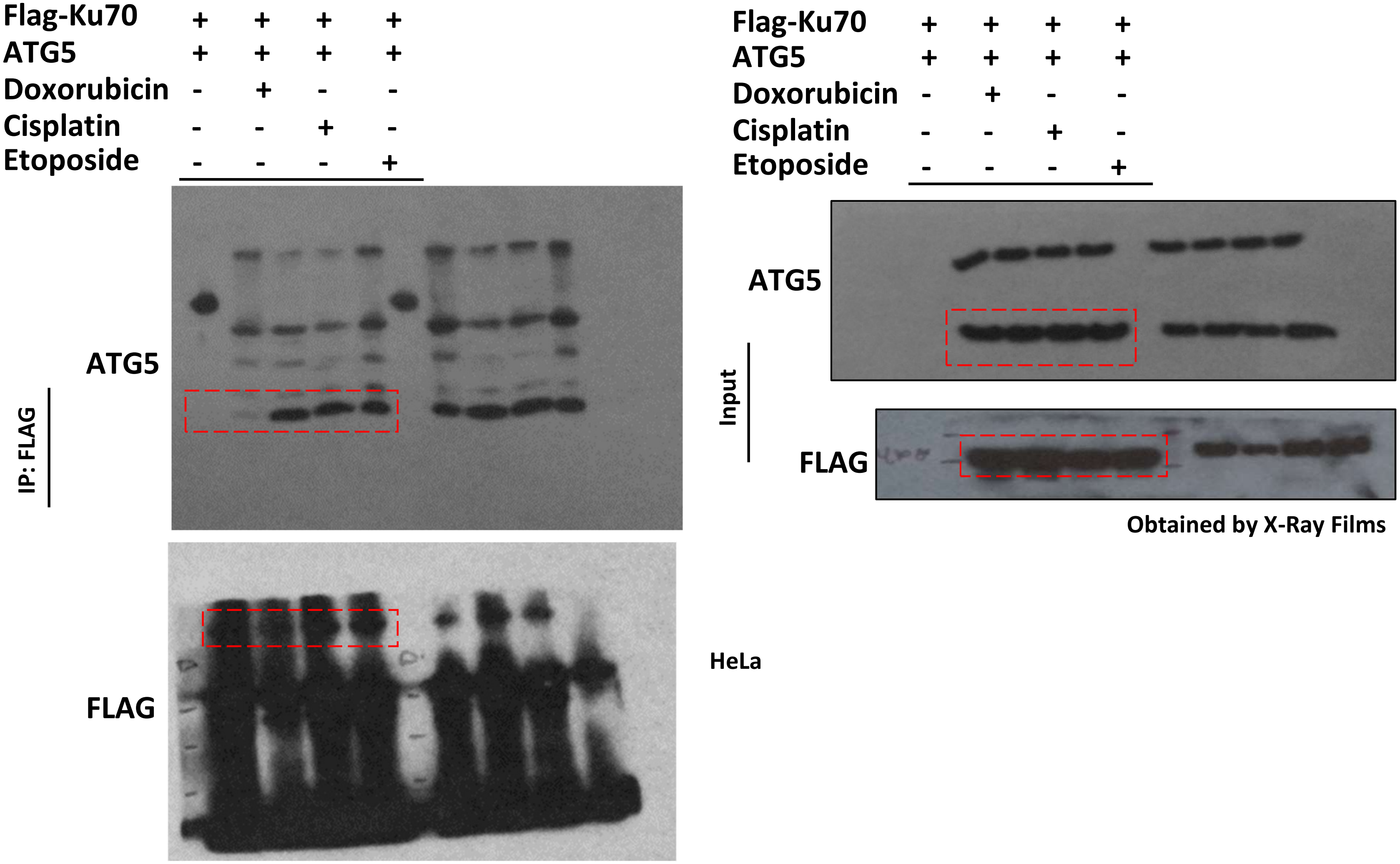
Flag-Ku70 + + + +
ATG5 + + + +
Doxorubicin - + - -
Cisplatin - - + -
Etoposide - - - +
ATG5
IP: FLAG
FLAG
HeLa
Flag-Ku70 + + + +
ATG5 + + + +
Doxorubicin - + - -
Cisplatin - - + -
Etoposide - - - +
ATG5
Input
FLAG
Obtained by X-Ray Films

Figure 1d

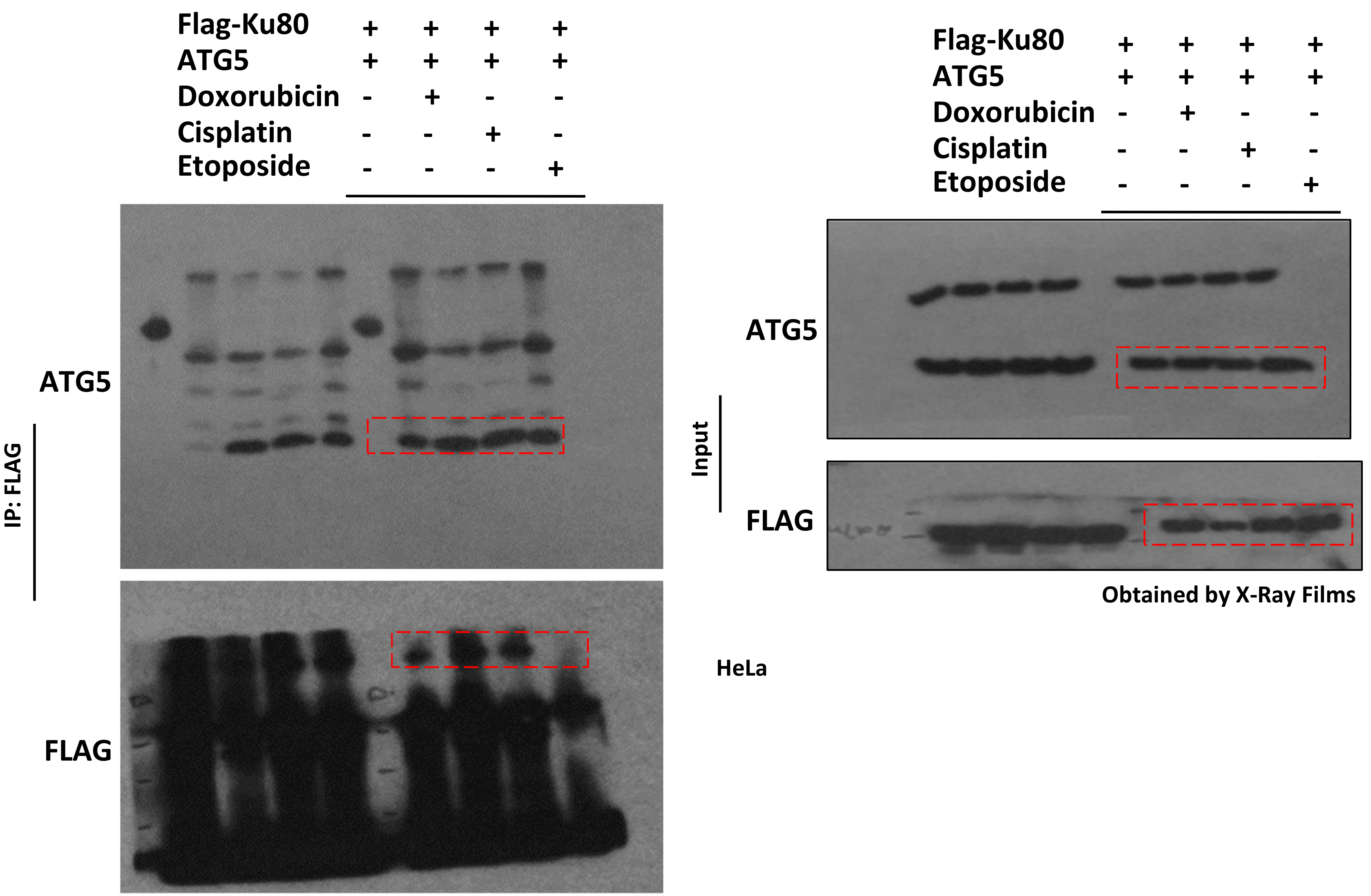
Flag-Ku80 + + + +
ATG5 + + + +
Doxorubicin - + - -
Cisplatin - - + -
Etoposide - - - +
ATG5
FLAG
IP: FLAG
HeLa
Flag-Ku80 + + + +
ATG5 + + + +
Doxorubicin - + - -
Cisplatin - - + -
Etoposide - - - +
ATG5
FLAG
Input
Obtained by X-Ray Films

Figure 2a

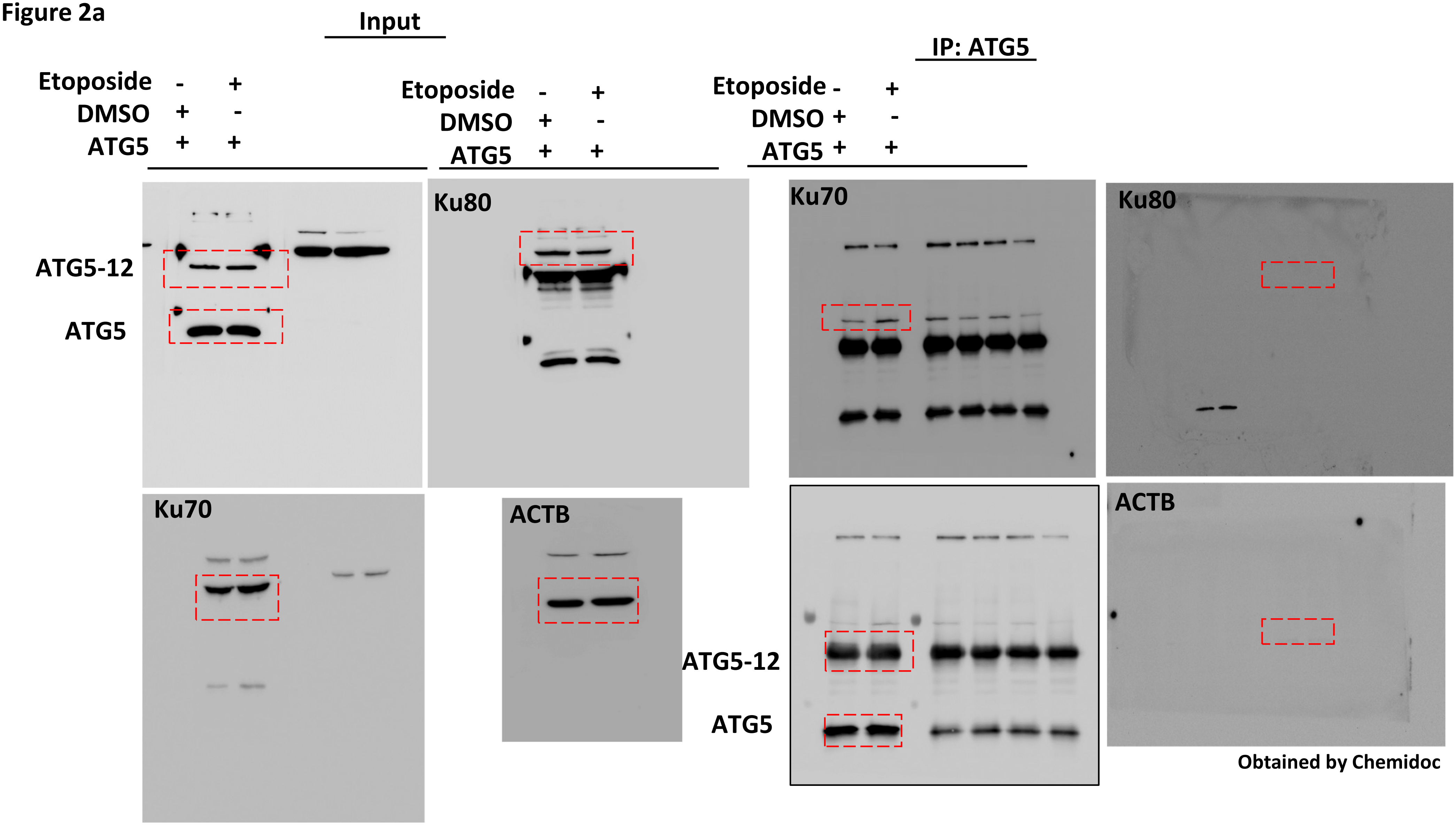

Obtained by Chemidoc

Figure 2b

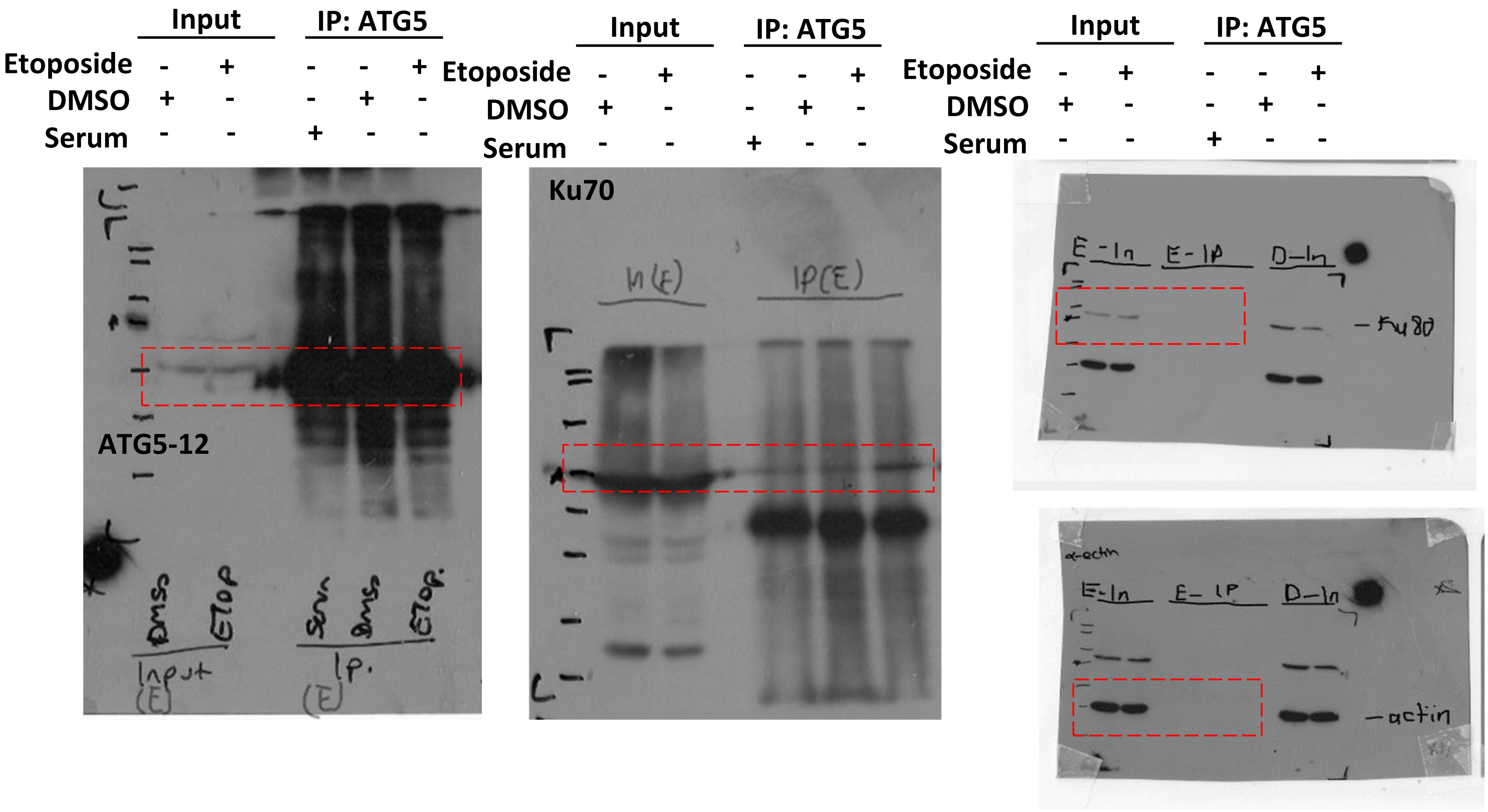

Obtained by X-Ray Films

Figure 2c

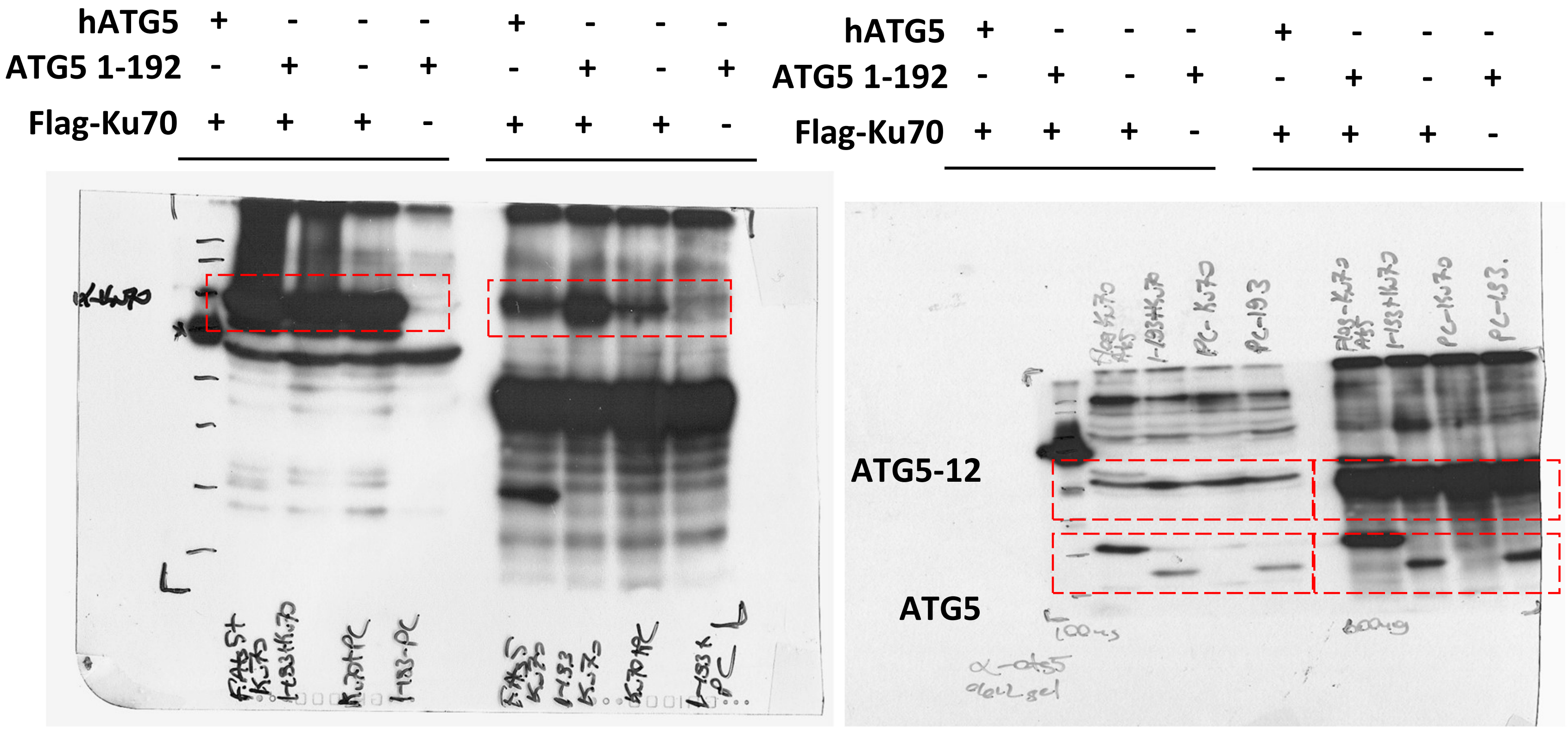

Obtained by X-Ray Films

Figure 3a-b

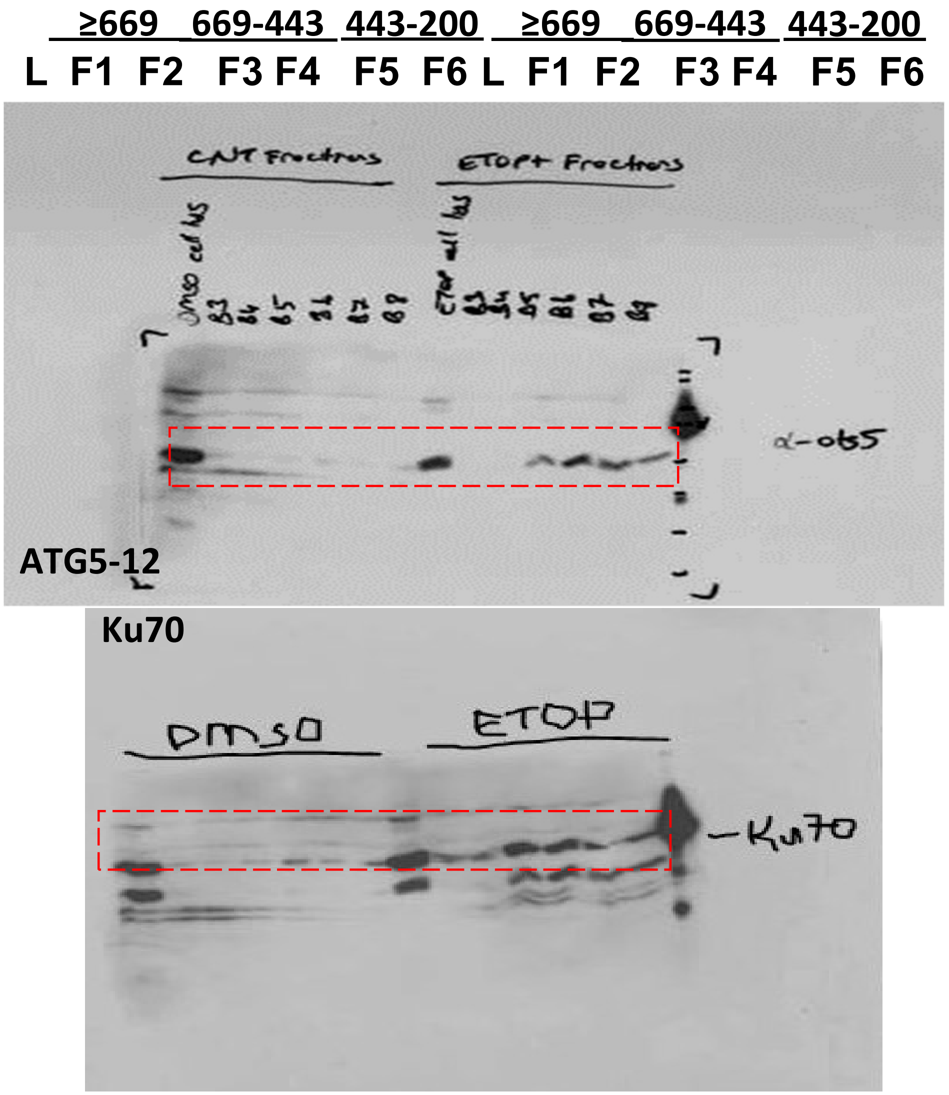

Figure 3c

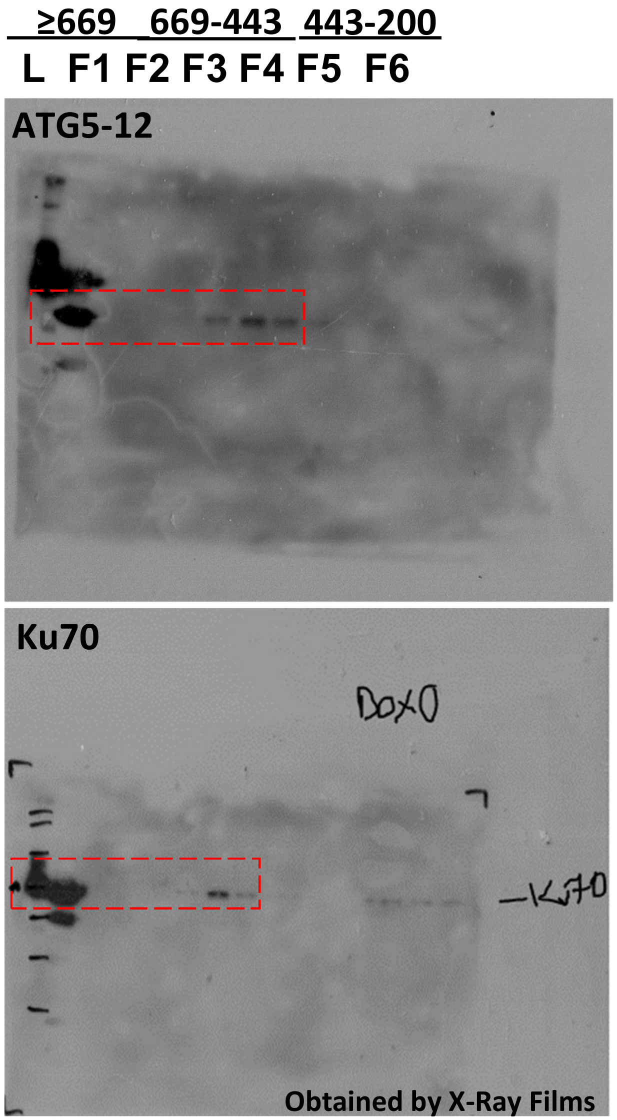

Figure 3f

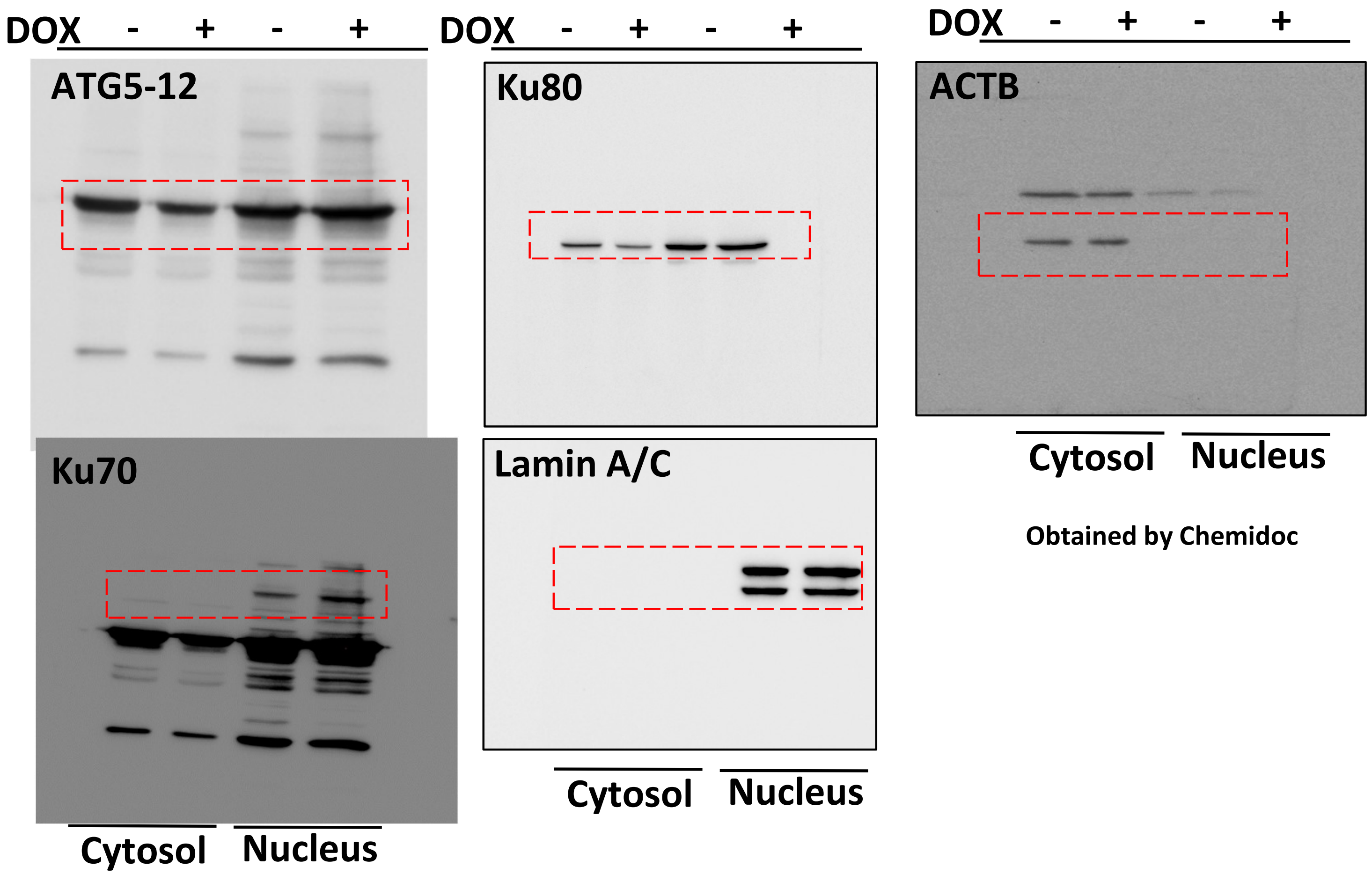

Obtained by Chemidoc

Figure 4c

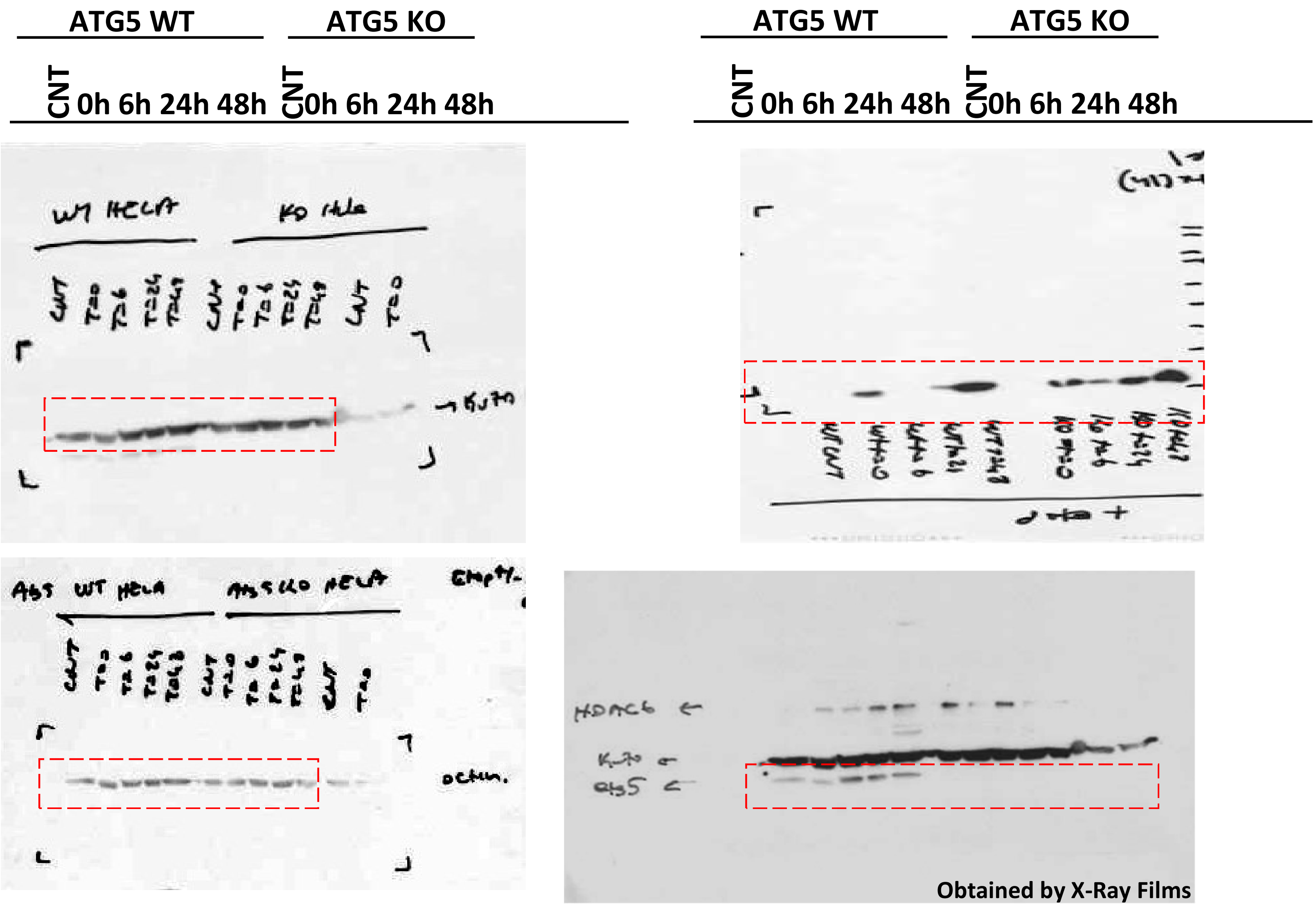
ATG5 WT
ATG5 KO
CNT 0h 6h 24h 48h
CNT 0h 6h 24h 48h
ATG5 WT
ATG5 KO
CNT 0h 6h 24h 48h
CNT 0h 6h 24h 48h
Obtained by X-Ray Films

Figure 4d

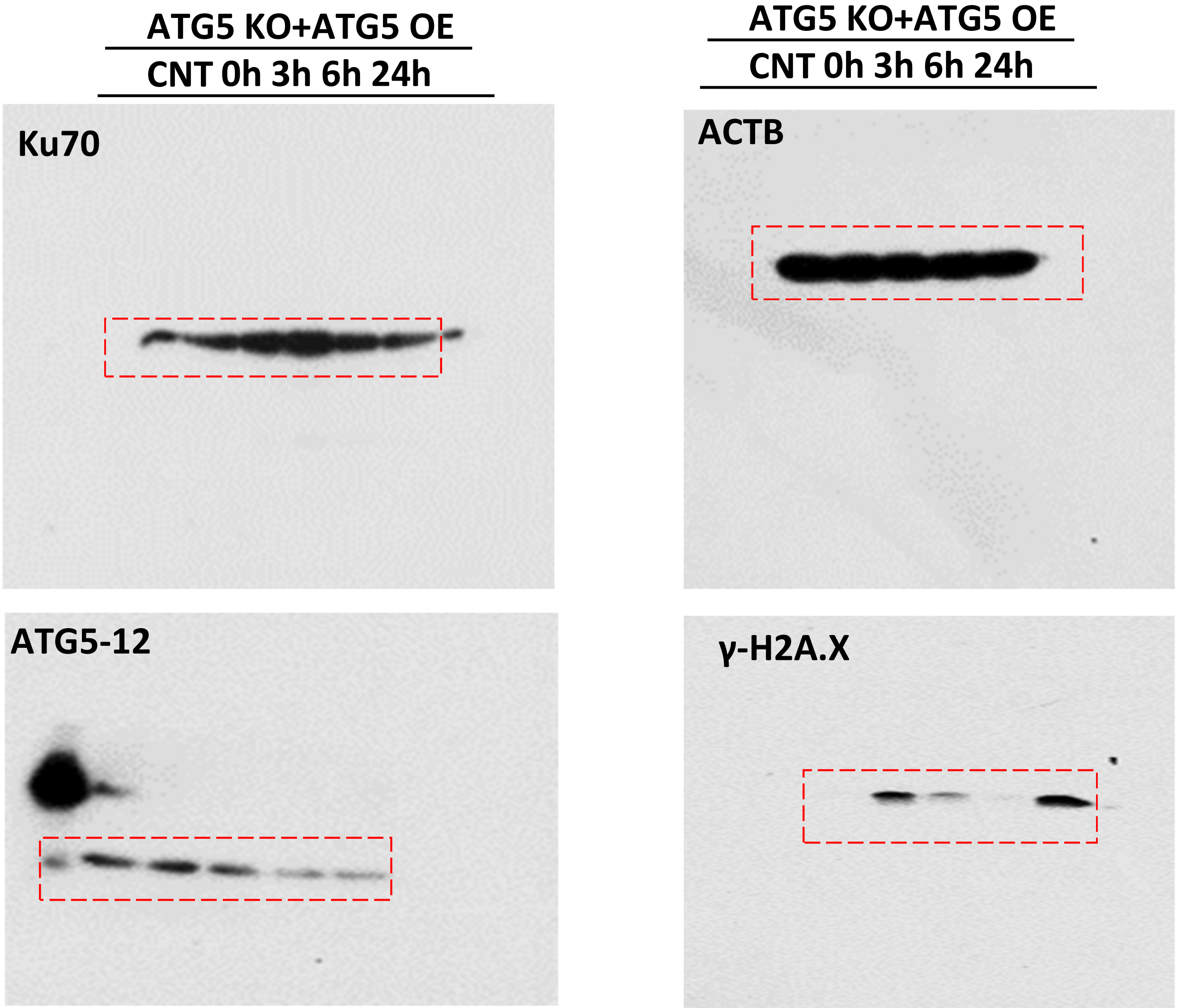

Obtained by X-Ray Films

Supp Figure 2a

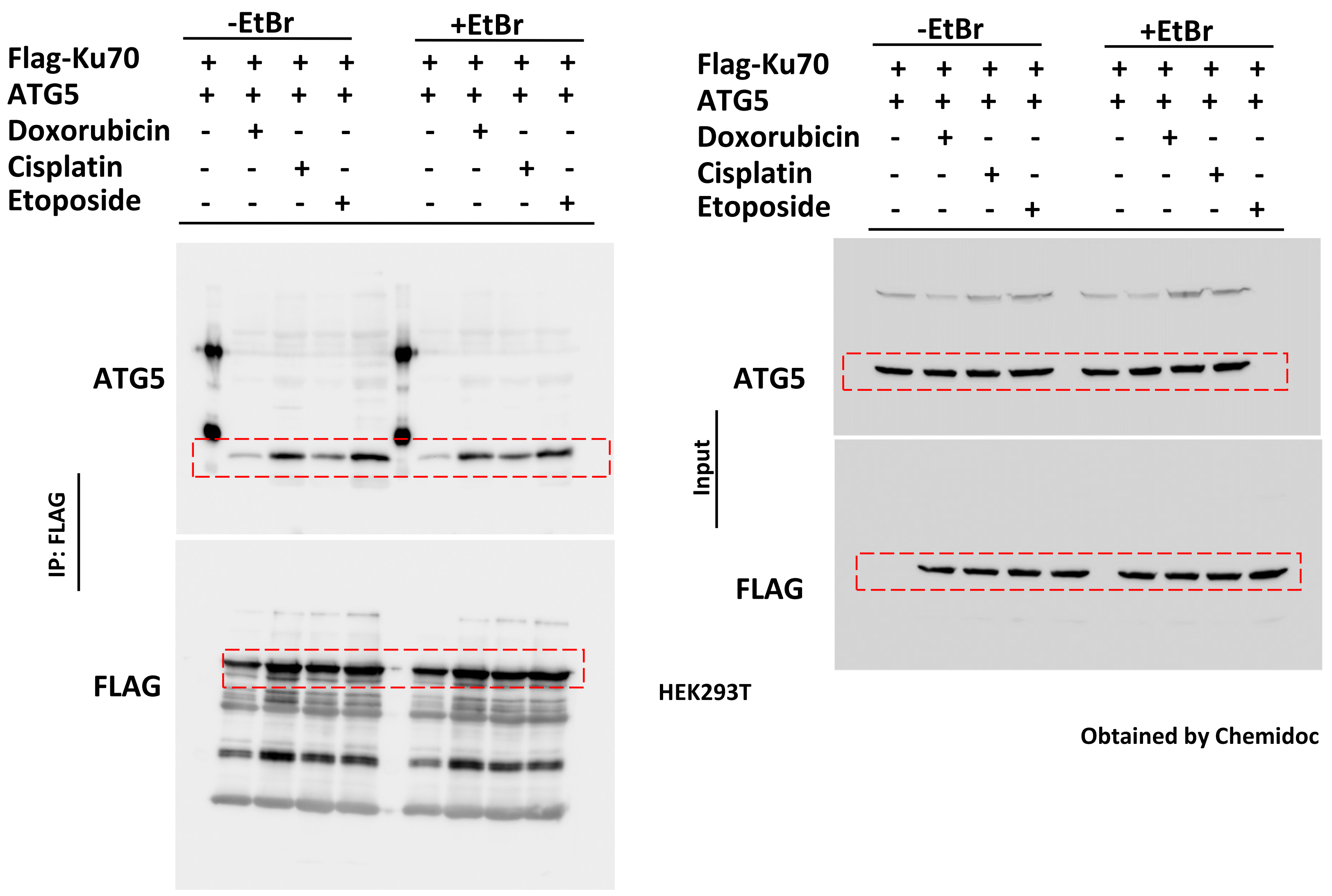

Supp Figure 2b

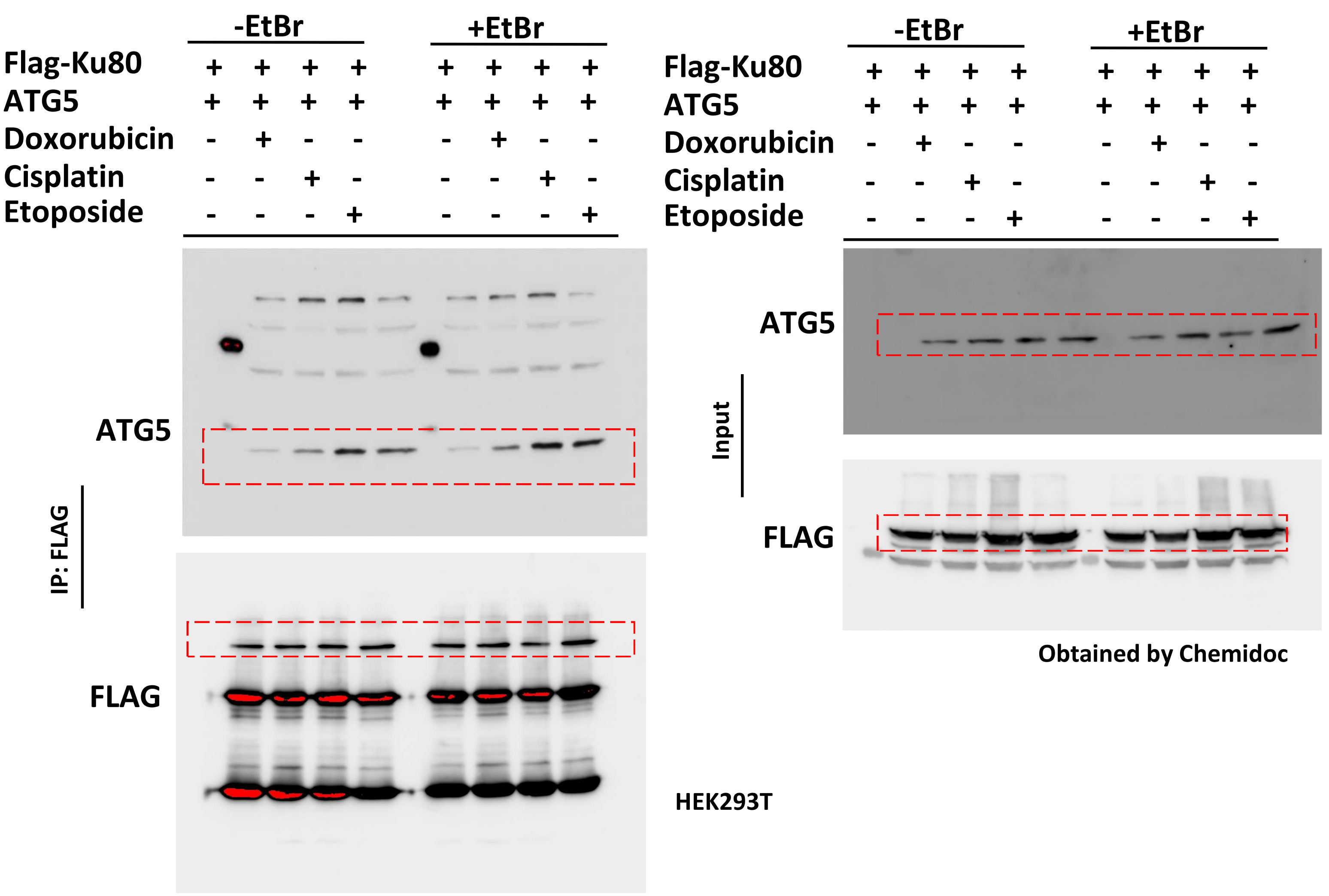

Supp Figure 2c

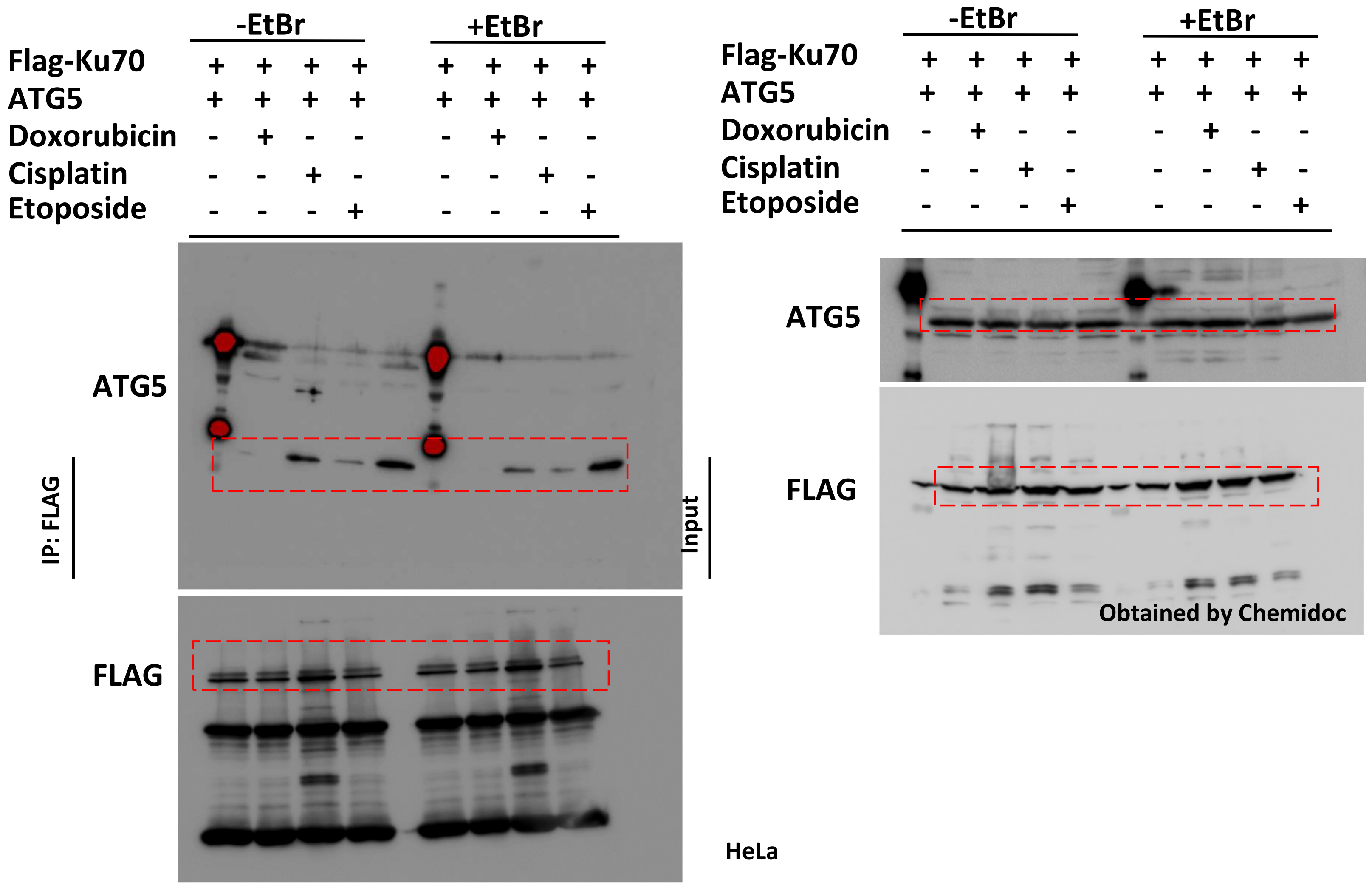

Supp Figure 2d

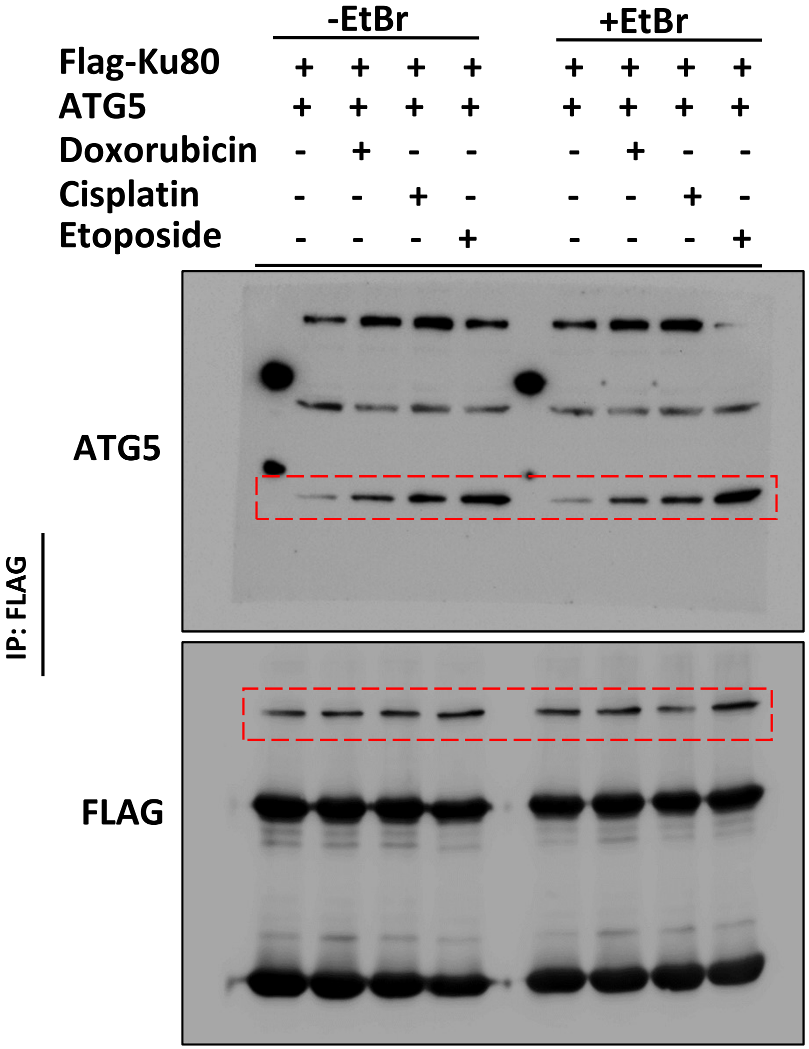

HeLa

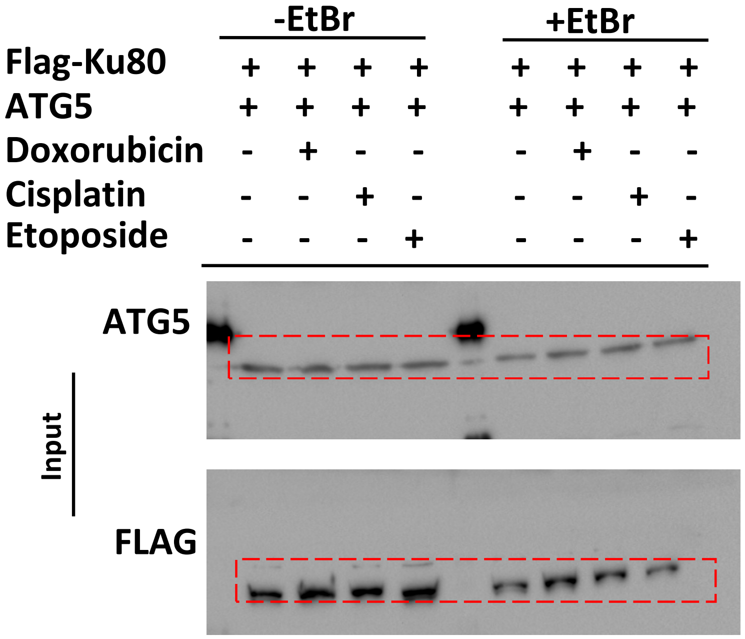

Obtained by Chemidoc

Supp Figure 3

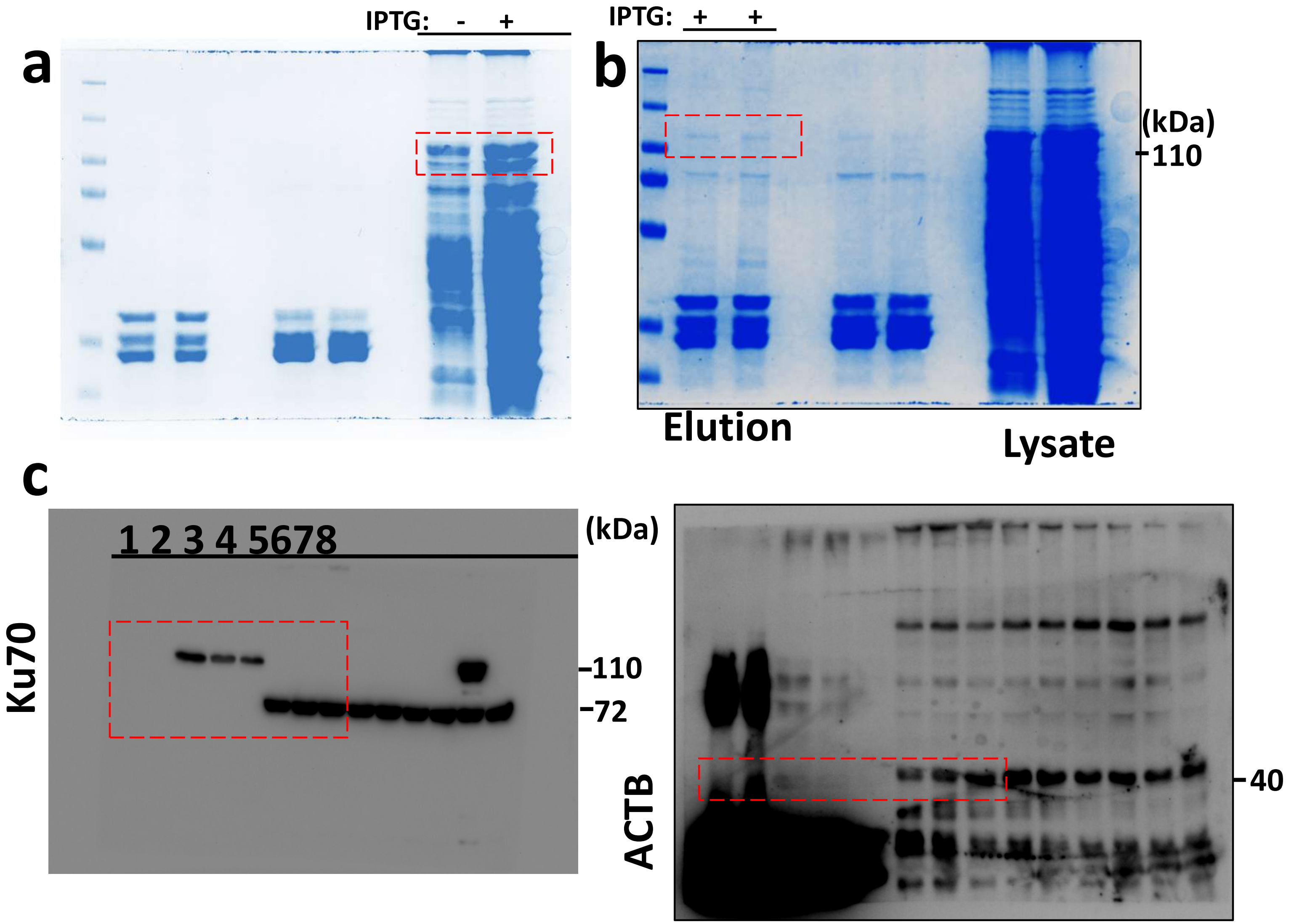

Supp Figure 5a

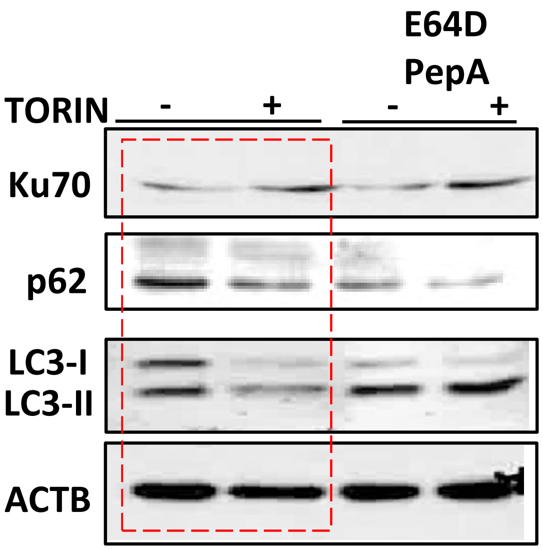

Obtained by X-Ray Films

Supp Figure 5b

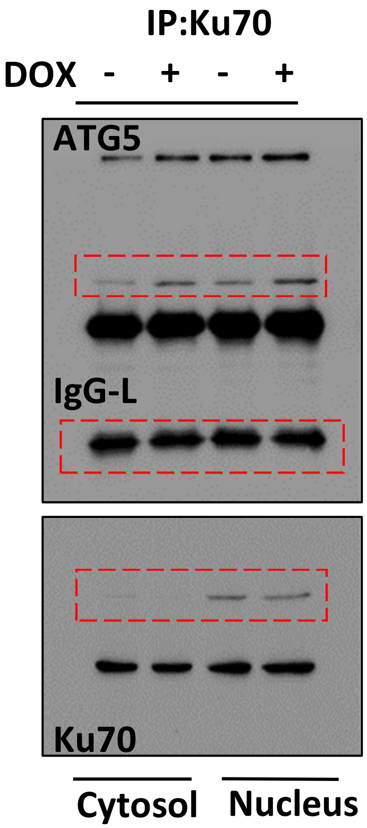

Obtained by Chemidoc

Supp Figure 6b

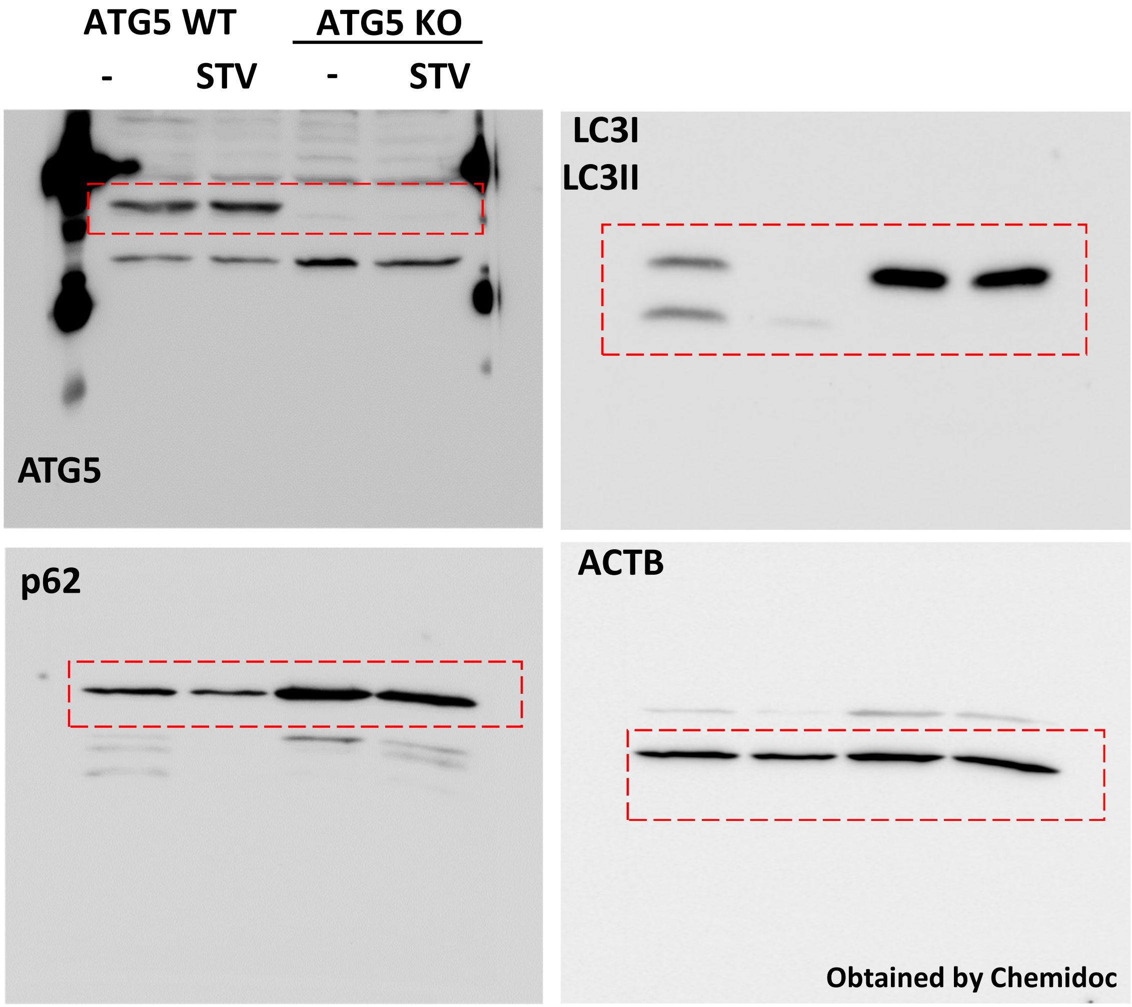

Supp Figure 7b

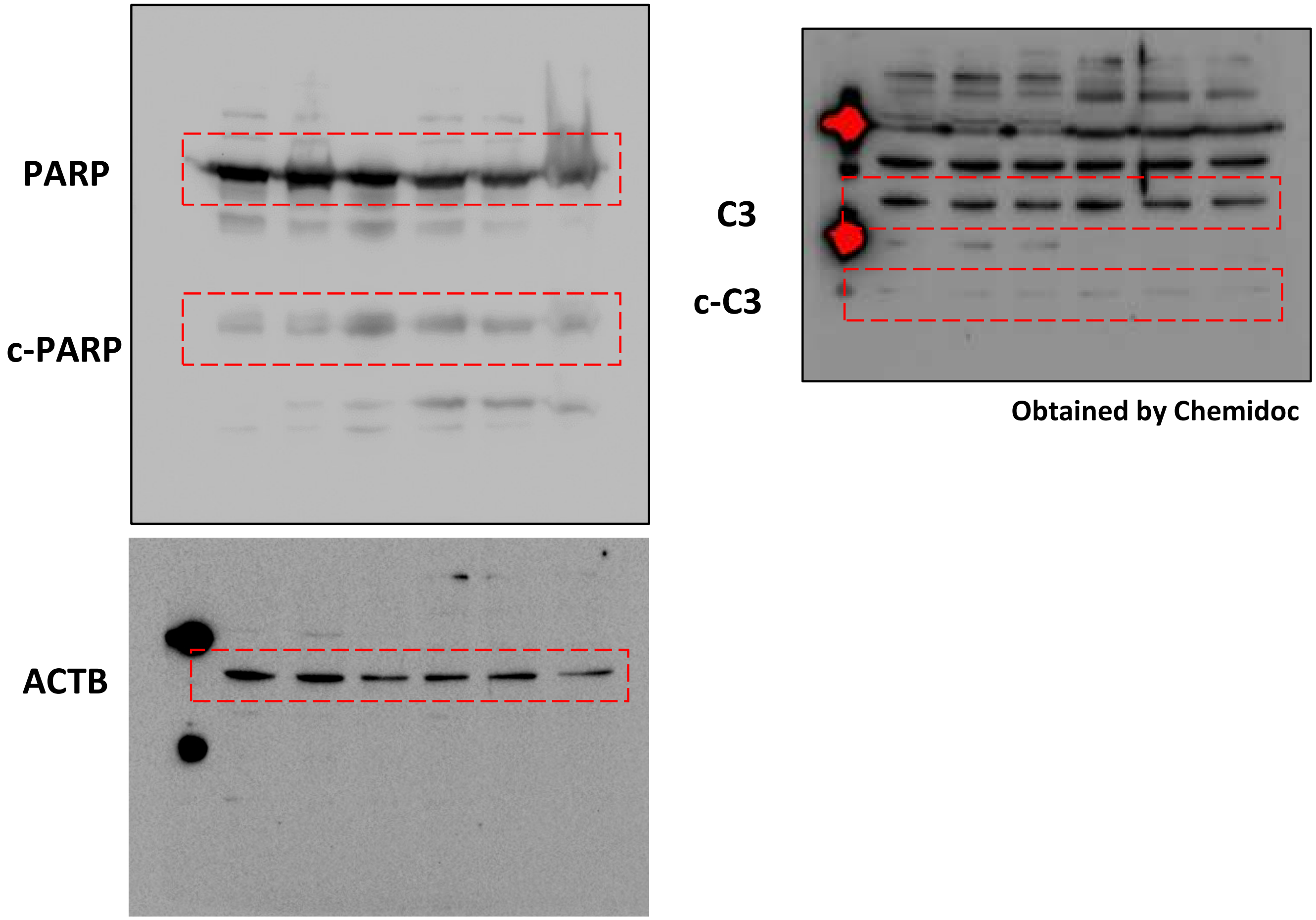

Supplement: Supplementary file 2 — Supplementary Information 2. [file 41598_2022_11704_MOESM2_ESM.pdf]
